# High intensity perturbations induce an abrupt shift in soil microbial state

**DOI:** 10.1038/s41396-023-01512-y

**Published:** 2023-10-09

**Authors:** Irene Cordero, Ainara Leizeaga, Lettice C. Hicks, Johannes Rousk, Richard D. Bardgett

**Affiliations:** 1 Department of Earth and Environmental Sciences, The University of Manchester, Michael Smith Building, Oxford Road, Manchester, M13 9PT UK; 2 Department of Community Ecology, Swiss Federal Institute for Forest, Snow and Landscape Research WSL, Zuercherstrasse 111, 8903 Birmensdorf, Switzerland; 3 Department of Biology, Lund University, Lund, Sweden

**Keywords:** Microbial ecology, Climate-change ecology, Grassland ecology

## Abstract

Soil microbial communities play a pivotal role in regulating ecosystem functioning. But they are increasingly being shaped by human-induced environmental change, including intense “pulse” perturbations, such as droughts, which are predicted to increase in frequency and intensity with climate change. While it is known that soil microbial communities are sensitive to such perturbations and that effects can be long-lasting, it remains untested whether there is a threshold in the intensity and frequency of perturbations that can trigger abrupt and persistent transitions in the taxonomic and functional characteristics of soil microbial communities. Here we demonstrate experimentally that intense pulses of drought equivalent to a 30-year drought event (<15% WHC) induce a major shift in the soil microbial community characterised by significantly altered bacterial and fungal community structures of reduced complexity and functionality. Moreover, the characteristics of this transformed microbial community persisted after returning soil to its previous moisture status. As a result, we found that drought had a strong legacy effect on bacterial community function, inducing an enhanced growth rate following subsequent drought. Abrupt transitions are widely documented in aquatic and terrestrial plant communities in response to human-induced perturbations. Our findings demonstrate that such transitions also occur in soil microbial communities in response to high intensity pulse perturbations, with potentially deleterious consequences for soil health.

## Introduction

Natural ecosystems are constantly exposed to natural fluctuations in environmental conditions and under such conditions they retain a stable equilibrium state, or quasi-stable state, characterised by minor fluctuations in community composition and function [1]. However, human-induced perturbations, including those related to climate change, can destabilise this dynamic equilibrium and potentially trigger a cascade of events that may lead to an abrupt change [2]. This is particularly relevant in a warmer world, where the speed of soil drying is increasingly higher [3] and multiple combined climate change factors interact [4]. Abrupt transitions occur when an ecosystem surpasses a certain threshold, which can have important consequences for ecosystem functioning [2]. Numerous studies have demonstrated the existence of abrupt changes in aquatic ecosystems [5, 6], terrestrial plant communities [7, 8], and the human gut microbiome in response to perturbations [9, 10]. However, abrupt transitions in soil microbial communities have so far received little attention [11], despite their fundamental role in terrestrial ecosystems, driving key processes of organic matter decomposition, nutrient cycling, and carbon and nutrient storage [12], which regulate ecosystem productivity. As such, abrupt shifts in soil microbial communities in response to perturbations may have significant implications for soil functioning, with consequences for ecosystem services, such as food production and climate regulation [13].

Soil microbial communities are increasingly challenged by perturbations associated with human-induced environment change, including intense “pulse” perturbations [14] caused by climate extremes (e.g., droughts, heat waves and floods), which are predicted to increase in frequency and intensity with ongoing climate change [15]. Microbial communities can withstand such pulse perturbations in different ways. In this context, resistance is defined as the degree to which microbial community attributes change in the face of a perturbation, and resilience as the rate at which they recover from it [11]. Thus, if the attributes (i.e., structure and function) of a microbial community stay stable following a perturbation, it is considered resistant [11, 16], whereas if its attributes change, but it recovers to its original configuration over time, it is considered resilient [11, 17]. Additionally, if the ecosystem processes remain stable whereas microbial community composition changes, this points to functional redundancy within the microbial community [18]. However, evidence is mounting that different microbial groups vary in their resistance and resilience to drought [19–21], and while intense droughts can de-stabilise soil bacterial networks, potentially rendering them more vulnerable to subsequent drought, soil fungal networks appear to be more resistant [22]. Frequent, recurring droughts can also lead to soil microbial communities becoming more resistant to drought [23–26], although repeated dry-wet cycles can induce shifts in the functional state of agricultural soils, measured as soil respiration [27]. Similarly, high intensity drought has been shown to cause abrupt shifts in peatland moisture characteristics [28] and plant-fungal interactions [29], although evidence of thresholds beyond which soil microbial communities have no resilience to drought, leading to abrupt shifts in their taxonomic and functional attributes, is lacking.

Here, we experimentally tested whether increases in the intensity and frequency of drought pulses can trigger an abrupt and persistent shift in the structural and functional attributes of natural grassland soil microbial communities. We also tested for the existence of a drought intensity and/or frequency threshold after which the soil microbial community shifts to a functionally and structurally different state. Moreover, we tested whether the bacterial and fungal communities showed any adaptation in their growth characteristics to the drying/rewetting cycle. To achieve this, we carried out an incubation experiment whereby we imposed a matrix of drought frequency and intensity treatments on a natural grassland soil. Microbial responses were assessed with a broad range of taxonomic and functional attributes of microbial communities over an extended period of time after returning soils to their original moisture condition. This enabled us to test for abrupt shifts in soil microbial communities that persisted under the same environmental conditions after the end of the perturbation. We hypothesised that soil microbial communities exposed to more intense and frequent droughts will show a lower resistance and resilience than those subject to milder, less frequent droughts. We also hypothesised that this lower resistance and resilience of microbial communities to more intense, frequent droughts will induce a shift in microbial taxonomic and functional state.

## Materials and methods

### Soil collection and experimental design

A pot experiment was designed to test the effects of drought intensity and frequency (3 levels each, full factorial including a well-watered control, Fig. S1), on the microbial communities of a natural grassland soil. Soil was collected from Selside, Yorkshire Dales (54.17 N, 2.34 W), from four independent plots (replicates). These plots correspond to the control plots in the experiment detailed elsewhere [30, 31]. The soils are part of the Malham Series of Eutric Endoleptic Cambisols; a clayey brown earth [32]. Chemical characteristics of the soil are shown in Table S1. The sampled soil was sieved and divided into pots. Pots were incubated at 18 °C, 30% air relative humidity, and kept at 65% water holding capacity (WHC), which correspond to ~40% volumetric water content. After 3 weeks of stabilisation, drought treatments were applied by reducing watering, reaching three selected drought intensity levels. A mild drought treatment (40% WHC, 23% volumetric water content) corresponded to average values of soil moisture during summer (June–August), an intermediate drought level (23% WHC, 14% volumetric water content) corresponded to common summer drought events (once every 4 years), and a high intensity drought level (11% WHC, 7% volumetric water content) corresponded to a once in a century drought in the studied ecosystem. Additional details can be found in SI methods. The drying period lasted for 2 weeks followed by 2 weeks of recovery, when pots were slowly rewetted to optimum moisture (65% WHC). All soils dried out at the very similar pace (Fig. S2a). Drought was repeated up to 3 times depending on the drought frequency treatment. Control pots were always kept at 65% WHC (Fig. S1).

Immediately after the last drought cycle, samples were collected to evaluate the resistance of the system. We consider the perturbation to be the entire drying/rewetting cycle, and thus, to evaluate the resistance we harvested the pots once the perturbation had ended (when all pots recovered to the same WHC, 11 days after the start of the rewetting, Fig. S2a). Additionally, pots were harvested over time, to evaluate the resilience of the system in the long term (1, 3, and 6 months after drought, Fig. S1), which covers the length of the typical growing season for the study site where soil was collected in the Yorkshire Dales (Met Office UK). During this whole period, pots were kept at optimum soil moisture (65% WHC). 40 extra pots were harvested 1 month after drought to evaluate the adaptation of microbial growth characteristics to drought. Total number of pots: 200.

### Microbial community structure

Soil samples were collected in Eppendorf tubes (approx. 0.25 g) and frozen at -80 °C immediately after sampling. DNA was extracted in frozen samples, without thawing, with PowerSoil DNA isolation Kit (Qiagen, Germany). DNA was sent to Macrogen sequencing service (Macrogen Inc., Korea), for sequencing on a MiSeq v3 (Illumina). Fungal diversity was evaluated by ITS2 sequencing, using the primer pair 5.8S-Fun and ITS4-Fun [33]. Bacterial diversity was evaluated by 16 S rRNA gene V3-V4 sequencing, with primers Bakt_341F and Bakt_805R [34]. Microbial community analysis was not done in samples from the 3 months after drought time point. Alongside the samples, three extraction blanks were included and a mock community sample for each primer pair: 19 strains genomic DNA even mix from Bakker lab for fungi [35], and MSA-1000 10 strain even mix genomic material for bacteria from the American Type Culture Collection (ATCC, Manassas, US).

Sequences were analysed using the DADA2 pipeline [36]. Taxonomic identification was performed by IDTAXA taxonomic classification method in DECIPHER [37] package using UNITE 7.2 reference database for fungi and SILVA release 138 database for bacteria. See SI methods for full details. After filtering, de-noising and refining steps, final databases contained 2760 amplicon sequence variants (ASVs) and 4,316,693 reads for fungi, and 7313 ASVs and 2,383,395 reads for bacteria. Mean sampling depth was 36,275 reads in fungi and 19,937 reads in bacteria.

### Soil functionality

Soil functionality was assessed by measuring soil enzymatic activities and different soil nutrient pools, as indicators, among others, of soil organic matter decomposition capacity, nutrient cycling capacity, soil fertility, available stocks of energy for microbial process, and soil carbon and nutrient storage capacity [38, 39]. *β*-glucosidase (GLC), cellobiohydrolase (CBH), xylosidase (XYL), *N*-acetylglucosaminidase (NAG), and acid phosphatase (PHO) were measured photometrically using *p*NP-linked substrate analogues [40]. Urease (URE) was evaluated by the production of ammonium after urea addition to soils, following the optimised high throughput method [41]. Phenoloxidase (POX) and peroxidase (PER) activities were measured photometrically by the oxidation of _L_-DOPA [42]. See SI methods for full details. All enzymes were measured in fresh soil, kept at 4 °C, within five days from harvest.

Different nutrient pools were measured by means of soil extractions with different extracting solutions depending on the nutrient and the pool of interest [43]. Dissolved organic carbon (DOC) and dissolved organic nitrogen (DON) were evaluated in water extracts and plant available nitrogen (ammonium and nitrate) were evaluated in 1 M KCl extracts. Plant available P was extracted with 2.5% acetic acid solution and total organic P (TOP) was estimated by evaluation of available phosphate before and after sample ignition at 550 °C for 4 h, and extracted with 0.5 M H_2_SO_4_ [44]. Microbial biomass nutrients were measured using fumigation–extraction techniques. Microbial C and N were measured after fumigation with CHCl_3_ and extraction with 0.5 M K_2_SO_4_ [45, 46]. Microbial P was estimated by fumigation with hexanol and extraction with anion-exchange membranes [47]. Microbial C, N, and P were calculated as the difference in C, N, and P between fumigated and un-fumigated samples, and they were converted to microbial biomass using k_EC_ factor of 0.35 for C [48], k_EN_ factor of 0.54 for N [45], and k_EP_ factor of 0.40 for P [49]. Microbial P was further corrected by sorption percentage using spiked samples. After extraction, N pools were measured in AA3 HR Auto Analyser (Seal Analytical, UK) while C pools were measured in 5000 A TOC-L analyser (Shimadzu, Japan). P pools were detected by molybdate colorimetry in a CLARIOstar plate reader (BMG Labtech, Germany). All soil nutrients were measured in fresh soil (except organic P), kept at 4 °C, within two weeks from harvest, including extraction and measurement of extracts. Nutrients were evaluated in duplicates, and reported values are the mean of those two analytical replicates. See SI methods for full details.

### Microbial functional adaptation to drying/rewetting cycles

Microbial adaptation to a subsequent dry-wet cycle was assessed using a two-tiered approach. First, the moisture dependence of microbial growth and respiration were assessed. To do so, soils were air dried under a ventilator until they reached constant weight. During the drying down, to assess how microbial functions were inhibited by lack of moisture, soils were subsampled every 2–3 h, and gravimetric water content, microbial growth and respiration were measured. Microbial growth rates were measured by radioisotope incorporation [50]. For bacterial growth, the rate of protein production was estimated using ^3^H-leucine incorporation into bacteria following homogenisation/centrifugation [51] with modifications as described previously [52]. Fungal growth rates were assessed by tracing ^14^C-acetate incorporation into the fungal-specific lipid ergosterol [53]. To measure soil respiration, 1.0 g of soil was weighed into 20 ml glass vials, which were purged with pressurised air, sealed with crimp caps and incubated. CO_2_ production was measured using a gas chromatograph equipped with a methaniser and a flame-ionisation detector. A logistic model was then fitted to the inhibition curves describing the relationship between microbial growth or respiration rates and moisture [54], and microbial tolerance to drought was estimated using IC_10_ values (moisture level at which the microbial function is inhibited by 10%), with lower values of IC_10_ indicating higher drought tolerance.

Second, air dried soils were rewetted to 60% WHC in order to evaluate microbial responses to rewetting. After rewetting, microbial growth rates, along with respiration, were measured with a high temporal resolution of approximately 6 h for a week (more frequent at the beginning and every 24 h afterwards, 12 time points in total), as previously described. Cumulative bacterial growth, fungal growth, and respiration during 1 week after rewetting was calculated. Furthermore, in all soils, bacterial growth exhibited a lag period of no growth before the growth rates started increasing exponentially, which has been previously observed [55]. These growth response patterns were therefore modelled using a Gompertz curve, which was then used to calculate the lag periods before the increase in growth rate [56].

### Statistical analyses

All statistical analyses were done in R v4.0 [57]. To evaluate microbial community structure, we investigated alpha diversity, ordination analyses, proportion of different taxa and functional guilds, and indicator species analysis. Resistance and resilience of soil functions were evaluated with a linear regression analysis between the value of the variable under drought and time after drought. The value of the intercept was used as a resistance index (RS), and the value of the slope as a resilience index (RL). Soil functional data (soil extracellular enzymes and nutrient pools) were analysed with a non-metric multidimensional scaling (NMDS) ordination analysis. Multifunctionality index was calculated with all the soil enzymatic activities, which represent the organic matter decomposition capacity of soils. The effects of drought intensity and frequency on all variables were analysed by linear mixed effect models (LME) with drought intensity and frequency as fixed factors and soil replicate as random factor. See SI methods for full details.

## Results

### Changes in microbial community structure

**Fig. 1:**
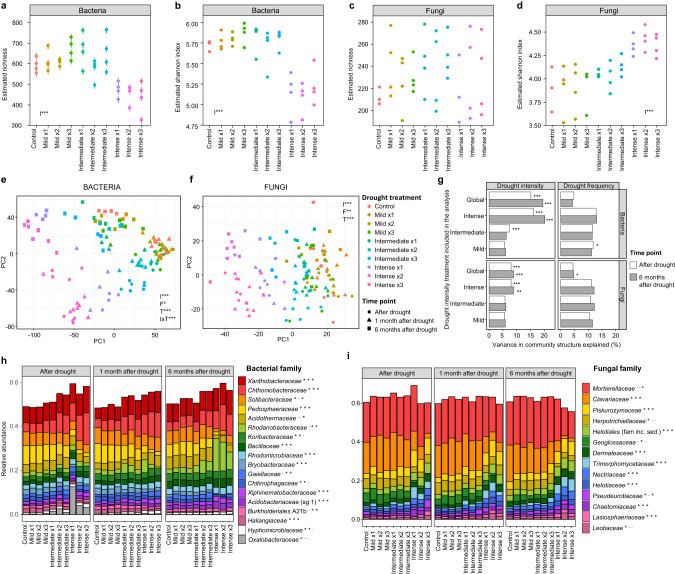
Effects of drought on soil microbial community structure. **a**–**d** Alpha diversity 6 months after drought (mean ± error of estimated values). **e, f** Community structure. The first two axes explained 17.3% (bacteria) and 5.4% (fungi) of the variance. **g** Percentage variance explained in PERMANOVA analyses including all the treatments (global) or just one drought intensity level and the control. **h, i** Relative abundance of microbial families. Data = mean (*n* = 4). sg subgroup. Significance of drought intensity (I), frequency (F), and time point (T) is shown (**a**–**g**): *p < 0.05, **p < 0.01, ***p < 0.001, or significance of drought treatment for each sampling time (**h, i**): ^-^*p* > 0.05, *p < 0.05.

Our data show that intense drought (~11% WHC), simulating a once in a century drought event in England, had a profound and long-lasting impact on soil microbial community structure and diversity, despite environmental conditions being returned to their original state (optimum moisture, 65% WHC). Six months after the end of the high intensity drought, bacterial diversity (Fig. 1a, b) was still reduced, while fungal Shannon diversity was significantly higher than in the non-droughted control treatment (Fig. 1d), despite no effects on fungal species richness being detected (Fig. 1c).

Bacterial and fungal community structures were strongly affected by drought (Fig. 1e, f, Fig. S3a, b) and changes in microbial community structures observed after drought were exacerbated with time. This is exemplified in the proportion of variance in community structure explained by drought intensity (Fig. 1g), which increased after 6 months compared to immediately after drought, particularly under the most intense drought treatment. However, bacterial communities in soils subjected to intermediate intensity drought which simulated drought events in England occurring every 4 years, partially recovered (Fig. 1g), with less variance explained by drought treatments after 6 months than after drought, i.e., they were more similar to the control treatments after 6 months than at the end of the perturbation. Mild drought, which simulated common drought events occurring annually in England, had no detectable impact on microbial community structure. Drought frequency also significantly affected bacterial and fungal community structures (Fig. 1e, f), with communities of those soils exposed to more frequent drought pulses being more distinct from the non-droughted control soils than those subject to fewer drought events.

The proportion of different bacterial taxa observed in the soil communities was mainly affected by drought intensity and time, with some minor effects of drought frequency (Table S2). Bacterial phyla *Proteobacteria*, *Actinobacteriota*, and *Firmicutes* increased in relative abundance with drought intensity, while taxa affiliated with *Acidobacteriota*, *Bacteroidota*, and *Myxococcota* decreased (Fig. S3c). There was a clear shift in the bacterial community, persistent through time, from a community co-dominated by *Acidobacteriota* and *Proteobacteria* taxa in the control towards one dominated by *Proteobacteria* under high intensity drought. At family level, particularly significant were the increases in ASVs affiliated with *Xanthobacteraceae*, *Chthoniobacteraceae*, *Rhodanobacteraceae*, and a transient increase in *Oxalobacteraceae* after drought (Fig. 1h), and the decrease in the relative abundance of *Solibacteraceae* and *Pedosphaeraceae* with drought intensity (Fig. 1h). Seventy-five of the 86 indicator genera identified for bacteria in our database (Fig. S4a) were indicators for all of the groups except the high intensity drought, i.e. their abundance was significantly reduced under intense drought. Only the genera *Edaphobacter, Paenibacillus, Streptomyces, Sphingomonas*, and an unassigned genus in the family *Intrasporangiaceae* were more abundant under intense drought than the rest of the treatments, and the genera *Tumebacillus*, and two unassigned genera in the families *Microbacteriaceae* and *Micrococcaceae* were more abundant under intense and intermediate drought.

The relative abundance of main fungal phyla was mostly affected by drought intensity and time (Fig. S3e, Table S2). We observed an increase in *Ascomycota* and a decrease in *Mortierellomycota*. In particular, we observed an increase in relative abundance of taxa affiliated with *Piskurozymaceae*, *Helotiales*, *Trimorphomycetaceae*, and *Nectriaceae*, and a substantial decrease of *Mortierellaceae* and *Clavariaceae*, the two most abundant fungal families (Fig. 1i), which are both typical soil saprotrophs. In agreement with this, we observed a significant decrease with drought treatments in the relative abundance of fungal taxa considered as saprotrophs (Fig. S3g). Fungal indicator genera (Fig. S4b) were mostly identified for the control + mild + intermediate drought group (17 out of 20), with no genus indicator for the most intense drought.

Bacterial diversity and community structure were correlated with a decrease in soil pH at the last sampling point (Fig. S5a–c), which was associated with increased nitrate ion concentrations at this sampling time (Fig. S5f). Fungal diversity was only marginally affected by soil pH (Fig. S5d,e).

### Changes in microbial community function

**Fig. 2:**
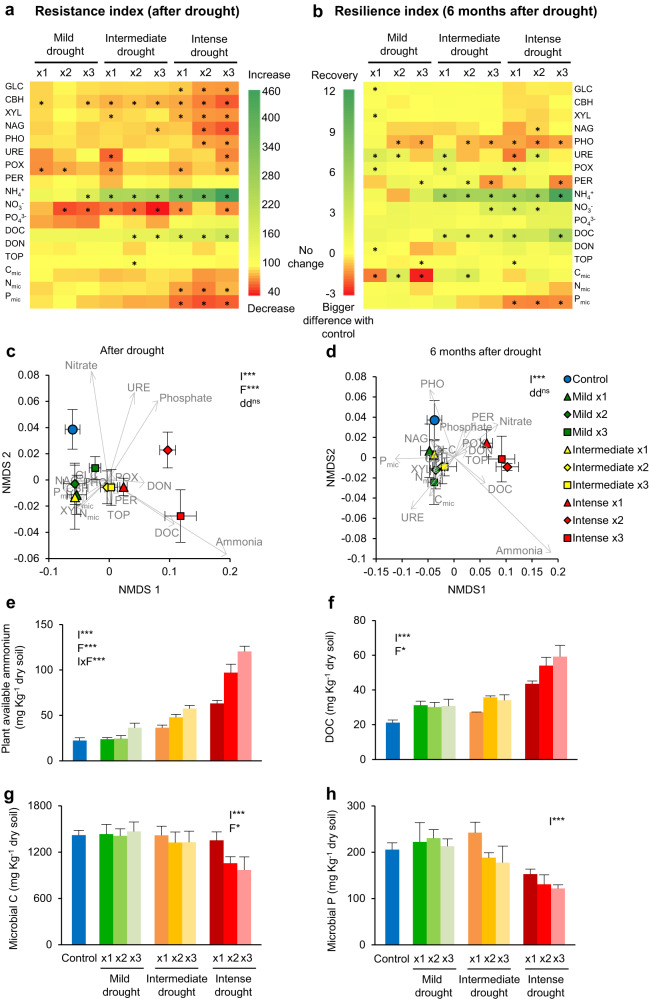
Effects of drought on soil function. **a, b** Resistance and resilience indexes. Asterisks indicate significant change (value ≠ 0, *p* < 0.05). **c, d** Functional structure of soils. Mean ± SE (*n* = 4). Significance of PERMANOVA analysis evaluating the effects of drought intensity (I) and frequency (F) is shown: *p < 0.05, **p < 0.01, ***p < 0.001. Differences in data dispersion (dd) among groups is also shown (^ns^ non-significant). GLC: *β*-glucosidase, CBH: cellobiohydrolase, XYL: xylosidase, NAG: *N*-acetylglocasiminidase, PHO: acid phosphatase, POX: phenoloxidase, PER: peroxidase, URE: urease, DOC: dissolved organic carbon, DON: dissolved organic nitrogen, TOP: total organic phosphorus, C_mic_: microbial carbon, N_mic_ : microbial nitrogen, P_mic_ : microbial phosphorus. **e**–**h** Effects of drought on selected soil nutrients after drought, summarised by drought intensity and frequency (x1: 1 event, x2: 2 events, x3: 3 events) treatments. Significance of linear mixed models evaluating the effects of drought intensity (I) and frequency (F), with soil as random factor, is shown in each graph. Values = mean ± standard error, *n* = 4.

After the end of drought, when soils were rewetted to the level of the control, seven of the eight extracellular enzyme activities evaluated were significantly reduced by drought intensity and/or frequency, displaying a low resistance to drought (Fig. 2a, Fig. S6a–h, Table S3). Phenoloxidase (POX) activity was slightly reduced by all drought treatments compared to the control, with no significant effect of drought intensity or frequency levels (Fig. 2a, Fig. S6g). Only the activity of peroxidase (PER) was not affected by drought (only a marginal effect of drought frequency) (Fig. S6h). The effects of drought intensity on soil enzymes were still detectable after six months (Fig. S6i–p), with very little or no resilience, or even a stronger reduction of their activity than immediately after drought (phosphatase) (Fig. 2b).

Soil nutrients were highly affected by drought, particularly available ammonium and dissolved organic carbon (DOC), with a significant increase due to drought intensity and frequency and an interaction between them: the more intense the drought, the bigger the effect of drought frequency on their concentration (Fig. 2e, f, Fig. S7a–f, Table S3). On the other hand, soil nitrate concentrations were reduced with most drought treatments compared to the control, showing a low resistance (Fig. 2a). After six months, this big ammonium and DOC flush had mostly disappeared and in turn, nitrate levels significantly increased (Fig. 2b, Fig. S7k–p). Drought had no significant effect on the resistance and resilience indexes of available phosphate (Fig. 2a), but there was a significant increase in available phosphate in the most intense and frequent drought treatment when evaluating the raw data after drought, instead of the calculated indexes (Fig. S7c). Microbial biomass C, N, and P were reduced by increasing drought intensity (Fig. 2a, h, i), and this effect was still detected after 6 months of returning soils to moisture levels of the control, with microbial P in intense drought treatments showing a stronger decrease with time (Fig. 2b, Fig. S7g–i, q–s, Table S3). Taken together, we observed that soils exposed to the most intense drought were in a significantly different functional state than the control soils even 6 months after the end of the perturbation (Fig. 2c, d).

### Microbial functional adaptation to drying/rewetting cycles

**Fig. 3:**
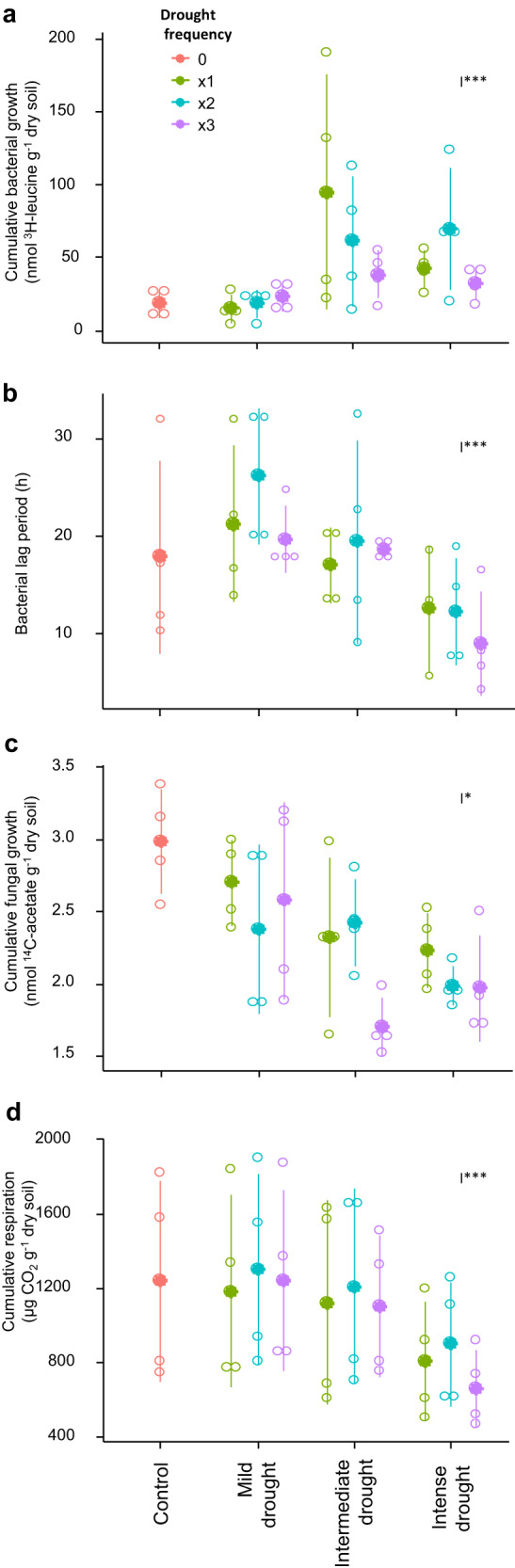
Adaptation to drought of microbial community traits. Growth responses to a further drying/rewetting cycle of soils with different drought history, one month after drought. **a** Cumulative bacterial growth, **b** bacterial lag period, **c** cumulative fungal growth, **d** cumulative respiration. Mean ± SD (*n* = 4). Significance of LME evaluating the effects of drought intensity (I) and frequency (F) is shown: *p < 0.05, **p < 0.01, ***p < 0.001.

Drought intensity had a legacy effect resulting in shorter lag times and higher cumulative bacterial growth (Fig. 3a, b) after rewetting, while cumulative fungal growth and cumulative respiration (Fig. 3c, d) significantly decreased with previous drought intensity. Fungi were more resistant to low moisture than bacteria, as shown by the lower IC_10_ (moisture level at which growth rate is reduced by 10%), but this was not significantly affected by previous drought treatments (Fig. S8a, b). Respiration showed reduced resistance to drought with previous drought intensity (Fig. S8c).

## Discussion

Our findings demonstrate that soil microbial communities are generally resistant to, and recover rapidly from, mild, infrequent droughts. However, we demonstrate a lack of resilience to high intensity drought, which triggers an abrupt and persistent shift in the soil microbial community to one of reduced structural complexity and impaired functioning. We also discovered that the functional characteristics of soil microbial communities can react to drought exposure, with exposure to high intensity drought inducing an enhanced ability of bacteria to recover growth rates following subsequent drought.

Bacterial and fungal communities were strongly and persistently modified by high intensity drought, with clear changes in diversity and community structure that persisted despite returning soils to their original moisture conditions. Consistent with previous studies, high intensity drought caused a reduction in bacterial diversity, which is indicative of reduced community resistance [22, 58]. Moreover, under high intensity drought, bacterial communities shifted from being co-dominated by *Acidobacteriota* and *Proteobacteria* to being dominated by *Proteobacteria*, with persistent increases in *Actinobacteriota* and *Firmicutes*, and a decrease in *Bacteroidetes* and *Myxococcota*. This community shift is consistent with previous reports on the drought tolerance of *Proteobacteria* [24], *Actinobacteria* [20, 23], and *Firmicutes* [59, 60], and drought sensitivity of *Acidobacteria* [20] and *Bacteroidetes* [58].

In contrast to bacteria, we observed an increase in fungal diversity under high intensity drought. This fungal community change was not associated with a change in species richness, but with an increase in evenness due to reduced abundances of the two dominant fungal taxa, *Mortierellaceae* and *Clavariaceae*, typical soil saprotrophs. These two families have been previously identified as drought sensitive [22, 61] and their decrease could be related to persistent changes in nutrient availability elicited by drought. Fungi are generally considered to be more resistant to drought than bacteria [22, 62] and several studies demonstrate a lack of drought effect on fungal communities [19]. In contrast, we observed a clear shift in the fungal community, which was still evident 6 months after returning droughted soils to their original, pre-drought moisture content. In our experiment, changes in community structure and the reduction in bacterial diversity were partially related to a decrease in soil pH, as previously demonstrated at a global [63] and local scale [64]. However, fungal diversity was only marginally related to soil pH, also in agreement with the literature [64, 65].

Microbial community shifts in response to high intensity drought were also associated with persistent changes in microbial functioning. Extracellular enzymes are not produced by a wide diversity of soil organisms [66], and, therefore, they do not reflect the whole soil community functionally. Additionally, part of the activity observed will come from stabilised enzymes within the soil matrix and not new enzymes produced by viable microbial cells [66]. Nevertheless, we observed a significant correlation between community composition and combined enzymatic activity in our experiment (Fig. S5g, h). High intensity drought effects on soil enzymes were still detectable after six months with very little or no recovery, manifesting a very low resilience and a persistent reduction of soil functional capacity, or functional regime shift. A reduction in soil enzymatic capacity with drought has been frequently reported [67], probably associated with microbial death and thus reduced enzyme production and reduced substrate diffusion that limits enzyme activity [68]. Enzymatic activities can also reflect the nutritional status of the microbial community and the existence of any particular nutrient limitation, as microbes invest in enzymes that minimise energy and nutrient costs and maximise benefits [68]. In agreement with this, enzyme activities were negatively correlated with available N and P (Fig. S5i-j). The lack of recovery of enzymatic capacity over time, even though soil nutrient returned to their original levels after rewetting, could be explained by consistently low microbial biomass 6 months after high intensity drought (Fig. S5l).

Increases in nutrient availability immediately after rewetting dry soils are likely explained by the death of microorganisms and increased availability of organic compounds [69, 70]. The fact that nitrate levels in our experiment significantly increased over time after rewetting, could be related to a high nitrification activity, where the highly abundant ammonium was transformed into nitrate [71]. Decreases in microbial biomass due to drought, as observed here under high intensity drought, have been widely reported [58, 72], although some authors observed an increase in microbial biomass under drought [73]. The observed constant decline of microbial biomass P over time since rewetting could be related to reduced phosphatase activity (Fig. S5m). This agrees with the study of Dijkstra and collaborators [74] which showed a strong reduction in P uptake by soil microbes during drought. However, a decrease in microbial biomass over time in bare soils is expected, as there is no additional C input from plants into the system.

As well as clear changes in soil microbial community structure and function elicited by drought, we also observed legacy effects of drought and adaptation of growth responses of bacterial and fungal communities when facing a further drying/rewetting cycle. The observed shorter lag times and higher cumulative growth for bacteria, in soils with a legacy of intense drought, are indicative of a faster recolonisation ability [75]. An increased and faster bacterial growth in soils with a history of drought could be a competitive adaptive strategy in soils exposed to frequent drought events [25, 26]. However, this could also be linked to changes in soil chemistry associated with the legacy of the different drought treatments. Although most of the nutrients released just after drought were already consumed at the time of the growth rate measurements (one month after drought), some were still elevated compared to control soils (Fig. S9), which could support bacterial growth. On the other hand, the reduced cumulative fungal growth could underpin the reduced abundance of the two dominant fungal families. This result contrasts with other studies where fungal growth was not affected by drought history [21, 76]. However, the fungal growth capacity in our study could have also been constrained by the high bacterial growth in the soils [77]. These effects on microbial growth were not dependent on the microbial biomass of soils before drying/rewetting (Fig. S5n, o). Cumulative respiration after rewetting seems to be driven by fungi, as it follows approximately the same pattern as cumulative fungal growth. This contrasts with some recent observations, where respiration was mostly driven by bacterial growth [21]. Alternatively, this decrease in cumulative respiration with previous drought intensity could be also related to resource availability. Previous high intensity drought led to a strong increase of DOC, which was mostly used one month after drought (Fig. S9c), and this likely depleted the soil carbon available to fuel a respiration peak after the additional drying/rewetting cycle. Reduced carbon availability in soils after drought has been previously reported [71], as well as less intense respiration peaks after repeated drying/rewetting cycles [25, 71].

Our results indicate that there are legacy effects of drought on soil microbial communities, matching recent studies. For example, pre-exposure to drought has been demonstrated to increase bacterial resistance [23, 24] and resilience [25] to subsequent droughts, although others have reported the opposite pattern, with previous drought reducing stability and diversity of microbial communities in the long term [22, 58]. We observed an increased recolonisation capacity of bacteria, albeit a lower tolerance to reduced soil moisture, while fungi showed a potential increased resistance to drought but with less cumulative growth upon rewetting. Thus, in this study system, there appears to be a trade-off between growth after rewetting, which can be interpreted as resilience, and resistance to low moisture. These strategy changes could be the result of a shift in the relative abundance of different taxa within the community or due to changes in individual taxa’s physiology or traits (evolution). In any case, the response of fast re-coloniser bacterial taxa appears to shape bacterial communities after drought, as they occupy niches left vacant after drought, conditioning community assembly afterwards [78].

**Fig. 4:**
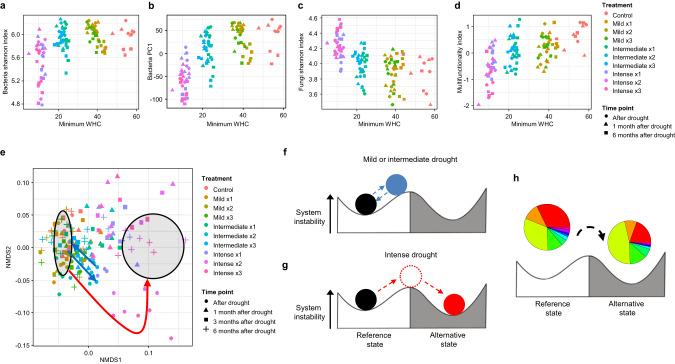
Drought threshold and potential shift to an alternative state in soil microbial communities. **a**–**d** Drought threshold: microbial community diversity, composition and multifunctionality depending on minimum water holding capacity (WHC) experienced by soils. **e** Functional capacity of soils evaluated by principal non-metric multidimensional scaling (NMDS) analysis with schematic representation of a shift to an alternative state. Mild or intermediate drought (blue arrows) moved the system from the reference state (circle on the left), but they bounced back. Intense drought (red arrow) moved the system further away, crossing a threshold, into an alternative state (circle on the right). **f**–**h** Diagrammatic representations of the effects of drought on the system, represented as a ball moving up and out of the stability basin. Mild or intermediate drought do not push the ball far enough, bouncing back to the reference state (**f**), while intense drought pushes the ball out of the reference state into a different stability basin, an alternative state (**g**), with distinct functionality and microbial community composition (**h**).

As discussed above, our findings provide experimental evidence that high intensity soil drying prevented microbial community resilience upon rewetting. Moreover, we observed a clear threshold of drought intensity level corresponding to a 30-year recurrent drought event in England (15% WHC, 9% volumetric water content; Fig. 4a–d). Below this moisture level, soil microbial communities were markedly and persistently restructured with impaired functioning, and they failed to recover over a period of 6 months, despite returning moisture levels to those of the control. The existence of this threshold is further supported by a previous study that demonstrated that below a threshold of 14% WHC, the growth pattern of bacteria upon rewetting changed significantly [79].

To further evaluate the abrupt and persistent microbial shifts in our experiment, we mapped microbial community composition and function with multivariate analysis, as it is a useful tool to visualise stability landscapes and regime shifts [80]. We observed a clear pattern in both community structure and function of soil microbial communities where soils subjected to intense drought occupy a distinct space separated from the control, which could potentially be interpreted as an alternative state of the system (Fig. 4e–h). Moreover, our experiment met most of the recognised criteria for detecting an alternative state [81]. First, we demonstrated the existence of two different microbial communities given the same environmental condition (i.e., at optimum soil moisture during the period after drought). Second, different intensities of drought, or the scale of pulse perturbation, were found to have contrasting effects on soil microbial communities, with mild and intermediate drought intensities showing reversible effects, but persistent changes in response to high intensity drought. Third, our experiment was conducted over an extended period of time, representing the typical length of the growing season in northern England, where the soil samples were collected. While it is unlikely that the identified microbial shifts are “stable”, we demonstrate that microbial communities subject to high intensity drought shift towards a different state that is distinct from its original one and from the non-droughted control soils. We therefore propose that the detected microbial shifts may reflect an alternative “transient” state [78] or simply an alternative state.

## Conclusion

Our findings demonstrate experimentally that while microbial communities can buffer mild, infrequent droughts, increasing the intensity and frequency of drought decreases soil microbial community resistance and resilience, and triggers an abrupt shift in soil microbial state. Moreover, we show that this abrupt shift in microbial state occurs at a threshold of <15% WHC - corresponding to a 30-year recurrent drought event in England - and is characterised by a pronounced and persistent reduction in microbial functional capacity, modified taxonomical composition of reduced complexity, and bacterial communities with a composition of functional traits that enable rapid recolonisation. Based on these finding, we propose that the detected microbial shifts may be indicative of an alternative microbial state after intense drought. However, caution is needed on extrapolating results from this laboratory study to real world settings and future studies are necessary to consider the role of extrinsic factors that might modify the vulnerability of soil microbial communities to perturbation-induced transitions to alternative states, such as the presence of plants, which can help the system recover after drought [82], or differences in nutrient availability and other soil abiotic properties [11]. Additionally, further experiments with longer time-scales and under settings closer to those found in the field are needed to better understand the responses of soil microbial communities to drought intensity and frequency, and how they vary under different climatic and edaphic conditions. Nevertheless, our results provide novel experimental evidence of a decreasing resistance and resilience of soil microbial communities as drought intensity increases, and identify a threshold for an abrupt and persistent shift in soil microbial state driven by high intensity drought, with potentially deleterious consequences for soil health.

### Supplementary information

Supplementary information

## Data Availability

Raw sequence reads are archived within the National Center for Biotechnology Information NCBI under project accession ID PRJNA1010014. Other data used in the study, including all functional data and processed ASV tables for bacteria and fungi, have been deposited in Figshare and are available at 10.6084/m9.figshare.24047193.v1.
