# Supplementary material for: High intensity perturbations induce an abrupt shift in soil microbial state
Source: ISME J. 2023 Oct 9;17(12):2190–9. doi: 10.1038/s41396-023-01512-y (PMC10690886; doi:10.1038/s41396-023-01512-y)
Supplement: Supplementary file 1 — Supplementary information [file 41396_2023_1512_MOESM1_ESM.pdf]

SUPPLEMENTARY INFORMATION

High intensity perturbations induce an abrupt shift in soil microbial state

Irene Cordero, Ainara Leizeaga, Lettice C. Hicks, Johannes Rousk and Richard D. Bardgett

SUPPLEMENTARY MATERIALS AND METHODS

Soil collection and drought treatments

Soils were collected in Selside, Yorkshire Dales (54.17 N, 2.34 W), from four independent plots (replicates). Turf was removed (2-3 cm), and topsoil collected from 3-10 cm depth. Main soil characteristics are shown in Table S1. Soils were sieved (4 mm mesh) and divided into pots (8 cm height, 8 cm diameter, and 170 ml volume), filled to mimic the mean field bulk density (0.82 g cm^{-3}). Pots were incubated at 18 ºC, 30% air relative humidity, and kept at 65% water holding capacity (WHC), which correspond to ~40% volumetric water content. After 3 weeks of stabilisation, drought treatments were applied by different watering regimes until they reached the target soil moisture: control treatments received 100% of the water loss, mild drought treatments received 2/3, intermediate intensity drought received 1/3 and intense drought received no water. Pots were watered every other day with autoclaved MiliQ water. Soil moisture was evaluated gravimetrically. Target soil moisture was calculated using historical data of soil moisture (1991-2020) in England, made available by the CCI SM project [28, 29]. Minimum annual values of soil moisture were fitted to an extreme value distribution (Gumbel distribution) with the package "extRemes" [30].

Drought lasted 2 weeks followed by 2 weeks of recovery when pots were slowly rewetted to optimum moisture (65% WHC), by adding the appropriate amount of water (by weight). Drought was repeated up to 3 times depending on the drought frequency treatment. Control pots were always kept at 65% WHC. Fig. S1a summarizes the evolution of WHC. WHC capacity was affected by the drought treatments (Fig. S1b), so after each drought cycle, WHC was re-evaluated. The decrease of moisture

retention capacity of soils after repeated drought has previously been documented (4, 5).

Microbial sequences analysis

The quality of sequences was assessed using FASTQC (9). Primers were removed using cutadapt (10) (fungi) or obitools (11) (bacteria). Low quality areas at the end of the reads were trimmed using truncLen= c(275,200) (fungi) or truncLen= c(260,180) plus 10 additional nucleotides at the beginning of forward reads (bacteria). Sequences were analysed using the DADA2 pipeline (12) with default parameters, except maxEE for fungi which was set to 4 in each direction. After filtering and de-noising steps 77% (fungi) or 44% (bacteria) of the reads were retained, and 4,227 (fungi) or 16,924 (bacteria) amplicon sequence variants (ASV) were identified. Taxonomic identification was performed by IDTAXA taxonomic classification method in DECIPHER (13) package using UNITE reference database (sh_general_release_dynamic_s_01.12.2017.fasta) for fungi and SILVA database (SILVA_SSU_r138_December2019.RData) for bacteria. Database was refined by four consecutive steps. 1) Renormalization to counteract the problem of tag-switching (14): for the abundant ASVs (≥ 100 reads), eliminate the reads of the samples corresponding to a cumulative frequency of less than 3% for each particular ASV. 2) Lulu algorithm to reduce the number of erroneous ASVs and achieve more realistic biodiversity metrics (15), setting the minimum ratio at 2 for fungi and minimum match at 99% for bacteria, and the rest parameters set as default. The election of these parameters was based on the results of the mock community sample. 3) Minimal abundance filtering, by removing any reads that represent < 0.02% abundance in each sample. 4) Blank correction, where ASVs were removed if the total abundance in

blanks divided by the total abundance in sample was greater than 10%, in each particular ASV. Finally, only fungi or bacteria sequences were retained. For fungal database, other eukaryotic reads that were removed represented the 0.01% of the reads. For bacteria, taxa unclassified at domain level represented the 2.83% of the reads, Archaea reads represented the 0.004% and chloroplast sequences represented the 0.01%. All of them were removed. Final databases contained 2,760 ASVs and 4,316,693 reads for fungi, and 7,313 ASVs and 2,383,395 reads for bacteria. Sampling depth varied from 25,873–53,007 (mean 36,275) reads in fungi and 12,595–58,150 (mean 19,937) reads in bacteria, and it was equally distributed among drought treatments. Rarefaction curves were plotted using the *rarecurve* function in "vegan" (16), and these indicated that sampling depth was sufficient for all samples, plateauing at around 7,500 reads.

Soil enzymes

β-glucosidase (GLC), cellobiohydrolase (CBH), xylosidase (XYL), *N*-acetylglucosaminidase (NAG) and acid phosphatase (PHO) were measured photometrically according to Jackson *et al*. 2013 (17), with modifications. 3.75 g of sieved soil was suspended in 5 mL of sodium acetate buffer (50 mM, pH 5.0). 150 µl of soil slurry were introduced into a 96-well deepwell block and mixed with 150 µl of a saturating substrate solution: 25 mM *p*NP-*β*-glucopyranoside for GLC, 2 mM *p*NP-*β*-D-cellobioside for CBH, 25 mM *p*NP-*β*-xylopyranoside for XYL, 5 mM *p*NP-N-acetyl-*β*-D-glucosaminide for NAG and 5 mM *p*NP-phosphate disodium salt hexahydrate for PHO. Plates were incubated at 18 ºC for 0.5 h (PHO), 1.5 h (GLC), 3.5 h (XYL and NAG) or 4 h (CBH) under continuous shaking. Blocks were centrifuged (2900 *g*, 5 min), 100 µl of the supernatant pipetted into transparent 96-well plates and mixed with

200 µl of 50 mM NaOH solution. Absorbance was measured at 405 nm in a plate reader (EZ400 Research, Biochrom, Germany) and corrected for soil and substrate colouration. Reported activity is the mean of four analytical replicates.

Urease (URE) was measured by the optimised high throughput method (18) without modifications. Briefly, 4 g soil was suspended in 10 ml of sodium acetate buffer (50 mM, pH 5.0) to create a slurry. 0.25 ml of soil slurry was extracted under continuous stirring and placed into deep-well blocks. Each well received 0.1 ml of 80 mM urea solution. Substrate blanks (only urea solution and buffer, no soil slurry) and soil controls (only soil slurry and buffer, no urea solution) were added. Blocks were incubated at 18 °C for 2 h under continuous shaking. After incubation, 1 ml of 2 M KCl was added to each well (to extract available ammonia) and blocks were shaken for a further 30 min. Blocks were then centrifuged (2900 *g*, 5 min), and 75 µl of the supernatant were pipetted into transparent 96-well plates and mixed with 75 µl of water. Ammonia concentration was evaluated by Berthelot reaction (Krom, 1980), and absorbance at 650 nm was measured in a microplate reader (EZ400 Research, Biochrom, Germany). Urease activity was expressed as µg $N\text{-}NH_4^+$ h^{-1} g^{-1} dry soil) and reported activity is the mean of four analytical replicates.

Phenoloxidase (POX) and peroxidase (PER) activities were measured photometrically following Sinsabaugh & Linkins 1988 (19) method, with modifications. 0.25 g of soil were suspended in 25 ml of sodium acetate buffer (50 mM, pH 5.0). 0.4 ml of soil slurry were extracted under continuous shaking and mixed (1:1) with a 20 mM L-3,4-dihydroxyphenylalanin (L-DOPA) solution in a deep-well block. Blocks were shaken for 10 min and centrifuged (2900 *g*, 5 min). 250 µl of the supernatant were pipetted into transparent 96-well plates. For peroxidase activity, wells additionally received 10 µl of a 0.3% H_2O_2 solution. Absorbance was measured at 450 nm in a

plate reader (EZ400 Research, Biochrom, Germany) at the starting time point (t_0), after 1.5 h (t_1 for PER) and after 20 h (t_1 for POX) at 18 ºC. Enzyme activity was calculated from the difference in absorption between the two time-points divided by L-DOPA molar extinction coefficient (7.9 μmol^{-1} (20)). For PER activity, POX activity was subtracted. Reported activity is the mean of three analytical replicates.

Nutrient pools

Dissolved organic carbon (DOC) and dissolved organic nitrogen (DON) were evaluated in water extracts (5 g soil in 28 ml MiliQ water) and plant available nitrogen (ammonium and nitrate) were evaluated in KCl extracts (2.5 g soil in 12.5 ml 1M KCl). In both cases, soil with extracting solutions were horizontally shaken at 200 rpm for 30 minutes and filtered through Whatman nº 42 filter papers (KCl extracts) or 0.45 µm syringe filters (H_2O extracts). N pools were measured in AA3 HR Auto Analyser (Seal Analytical, UK) while C pools were measured in 5000A TOC-L analyser (Shimadzu, Japan). Plant available P was extracted with acetic acid solution (1 g soil + 25 ml 2.5% acetic acid) and detected by molybdate colorimetry using AA3 HR Auto Analyzer (Seal Analytical, UK).

Organic P was estimated by evaluation of available phosphate before and after sample ignition (22). Two portions of dry soil (2 g) were weighed. One of them was burned at 550ºC for 4 h in a furnace. Afterwards, both samples were extracted with 50 ml 0.5 M H_2SO_4 by horizontally shaking at 120 rpm for 16 h. Extracts were filtered (Whatman No. 42) and phosphate was evaluated by the ascorbic acid method (22). After sample neutralization with NaOH, the colour reaction was carried out and the amount of blue was evaluated by absorbance at 880 nm in a CLARIOstar plate reader (BMG Labtech, Germany).

Microbial biomass C and N was measured using the fumigation–extraction techniques (23, 24). 2.5 g of fresh soil were fumigated with $CHCl_3$ for 24 h. Soluble C and N were extracted from the fumigated and from un-fumigated samples with 12.5 ml 0.5 M K_2SO_4. Soil + extracting solution were shaken and filtered (Whatman No. 42) and total C and N were analysed in TOC and AA, respectively. Microbial C and N flush (difference between fumigated and un-fumigated samples) were converted to microbial biomass using k_{EC} factor of 0.35 (25) and k_{EN} factor of 0.54 (23).

Microbial P was estimated with the hexanol fumigation and extraction with anion-exchange membranes method (26). Membrane strips (1 x 4 cm) were prepared by shaking in 0.5 M $NaHCO_3$. For each sample, three portions of fresh soil (2 g) were weighed into 50 ml tubes: unfumigated, fumigated and spiked samples. In each tube, 30 ml deionized water and three anion-exchange membrane strips were added. Fumigated samples received 1 ml of hexanol while spiked samples received 1 ml of a 20 mg ml^{-1} P solution. All tubes were shaken for 24 h. The membranes were then removed and rinsed with deionized water and phosphate recovered by shaking for 1 h in 20 ml of 0.25 M H_2SO_4, with detection at 880 nm by automated molybdate colorimetry using AA3 HR Auto Analyzer (Seal Analytical, UK). Microbial phosphorus were calculated as the difference between the fumigated and un-fumigated samples, corrected by the sorption percentage (spiked samples) and transformed using a k_{EP} factor of 0.40 (27).

Statistical analyses

All statistical analyses were done in R v4.0 (32). To evaluate microbial community structure, we investigated alpha diversity, community structure with ordination analyses, proportion of different taxa and functional guilds (only for fungi) and we

performed indicator species analysis. Due to the issues associated with using rarefied or relative abundance data for diversity tests and differential abundance analyses (33), different approaches or data transformations were applied for each analysis (specified below).

Alpha diversity was calculated estimating the unobserved diversity (34). Richness was estimated with “breakaway” package (35) and Shannon index with “DivNet” package (36). Community structure was explored with a variance stabilization transformation (VST) of data, using “DeSeq2” package (37). PERMANOVA analyses using Euclidean distance of transformed data were performed to evaluate differences among drought treatments, with soil as random factor, using *adonis* function in “vegan” package (16). To better visualise the effects of the drought treatments, a correction to eliminate the soil effect was performed with *removeBatchEffect* function in “limma” package (38). A PCA analysis was then performed in the corrected database using “vegan” (16). To predict functional guilds and trophic modes from the fungal taxonomic data we used FUNGuild (39). Only those assignments considered highly probable or probable were used, while possible assignments were discarded. Assignments with multiple and contradictory guilds to the same taxa were also discarded. In average, 29.1% of the taxa (774 ASVs) were assigned and used in this analysis. Of those, 82 were assigned to order level, 158 to family level and 534 to genus level. For relative abundance graphs, only taxa (phylum, order or family) with relative abundances >1% across all samples were plotted.

Indicator species analysis was performed using *multipatt* function in the “indicspecies” package (40) to identify bacterial and fungal taxa associated with drought and control treatments. We tested for indicator taxa depending on the intensity of drought, comparing the control treatment with the most intense drought and some

specific combinations (control+mild, control+mild+intermediate, and intermediate+intense drought). Prior to analysis, ASVs were agglomerated at genus level using *tax_glom* in “phyloseq” package (41), and libraries were normalised using cumulative sum scaling (CSS) (42), using *phyloseq_transform_css* function in “metagMisc” package (43). CSS-normalised abundance heatmaps were produced using *plot_heatmap* function in “phyloseq” (41).

Resistance and resilience of soil functions were evaluated with a linear regression analysis between the value of the variable under drought and time after drought (in weeks). We used relative data in percentage (i.e., value drought pot/value control pot × 100), and we performed individual analyses for each of the nine drought intensity and drought frequency combinations. The value of the intercept was used as a resistance index (RS), as it represents the relative difference of the drought treatment with the control just after drought. RS can be directly interpreted as the % change compared to the control treatment. Additionally, we used the value of the slope as a resilience index (RL). This value was adjusted depending on the direction of change after drought with the formula: if intercept < 100, then RL = slope; if intercept > 100, then RL = −slope. Thus, a positive RL indicates a recovery of the system; variables in drought pots are getting more similar to those in control pots. On the other hand, RL = 0 reflects no change (no resilience), with drought pots showing the same difference with the control ones that what it was at the end of the drought. Moreover, a negative RL can be interpreted as delayed response to drought or a continuous negative effect even when the perturbation has finished, as negative RL mean a bigger difference with the control over time than what it was at the end of the drought. RL values can be directly interpreted as percentage recovery/change per week.

Soil functional data (soil extracellular enzymes and nutrient pools) were analysed with a non-metric multidimensional scaling (NMDS) ordination analysis, performed with the function *metaMDS* in “vegan” (16), using relative data (value/average of the control at each sampling date). Multifunctionality index (44) was calculated with all the soil enzymatic activities, which represent the organic matter decomposition capacity of soils. Data were standardised by z transformation separately for each sampling time, which removed overall differences between harvest time points (45). Subsequently, the average of all standardised values was used as the multifunctionality index.

The effects of drought intensity and frequency in all variables were analysed by linear mixed effect models (LME) with drought intensity and frequency as fixed factors and soil replicate as random factor, with *lme* function in “nmle” package (46). To obtain a balanced design, control pots were removed from the analysis. Normality of the residuals and homoscedasticity were confirmed with Anderson-Darling test and Levene test, respectively. If needed, natural logarithmic or square root transformations were applied. If the assumptions were still not met, Kruskal-Wallis test was performed instead. *P* values after multiple comparisons (for bacterial and fungal taxa) were adjusted with the Benjamini-Hochberg adjustment (47, 48) using *p.adjust* function.

REFERENCES

1. W. Dorigo, *et al.*, ESA CCI Soil Moisture for improved Earth system understanding: State-of-the art and future directions. *Remote Sens. Environ.* **203**, 185–215 (2017).

2. A. Gruber, T. Scanlon, R. Van Der Schalie, W. Wagner, W. Dorigo, Evolution of the ESA CCI Soil Moisture climate data records and their underlying merging methodology. *Earth Syst. Sci. Data* **11**, 717–739 (2019).

3. E. Gilleland, R. W. Katz, extRemes 2.0: An Extreme Value Analysis Package in R. *J. Stat. Softw.* **72**, 1–39 (2016).

4. D. A. Robinson, *et al.*, Experimental evidence for drought induced alternative stable states of soil moisture. *Sci. Rep.* **6**, 20018 (2016).

5. K. Ahmadi, *et al.*, Effects of rhizosphere wettability on microbial biomass, enzyme activities and localization. *Rhizosphere* **7**, 35–42 (2018).

6. D. Taylor, *et al.*, Accurate estimation of fungal diversity and abundance through improved lineage-specific primers optimized for Illumina amplicon sequencing. *Appl. Environ. Microbiol.* **82**, 7217–7226 (2016).

7. D. P. R. Herlemann, *et al.*, Transitions in bacterial communities along the 2000 km salinity gradient of the Baltic Sea. *ISME J.* **5**, 1571–1579 (2011).

8. M. G. Bakker, A fungal mock community control for amplicon sequencing experiments. *Mol. Ecol. Resour.* **18**, 541–556 (2018).

9. S. Andrews, FastQC: A quality control tool for high throughput sequence data. http://www.bioinformatics.babraham.ac.uk/projects/ (2010).

10. M. Martin, Cutadapt removes adapter sequences from high-throughput sequencing reads. *EMBnet.journal* **17**, 10–12 (2011).

11. F. Boyer, *et al.*, OBITOOLS: A UNIX-inspired software package for DNA metabarcoding. *Mol. Ecol. Resour.* **16**, 176–182 (2016).

12. B. J. Callahan, *et al.*, DADA2: High-resolution sample inference from Illumina amplicon data. *Nat. Methods* **13**, 581–583 (2016).

13. E. S. Wright, Using DECIPHER v2.0 to analyze big biological sequence data in R. *R J.* **8**, 352–359 (2016).

14. O. S. Wangensteen, C. Palacín, M. Guardiola, X. Turon, DNA metabarcoding of littoral hard-bottom communities: high diversity and database gaps revealed by two molecular markers. *PeerJ* **6**, e4705 (2018).

15. T. G. Frøslev, *et al.*, Algorithm for post-clustering curation of DNA amplicon data yields reliable biodiversity estimates. *Nat. Commun.* **8**, 1188 (2017).

16. J. Oksanen, *et al.*, vegan: community ecology package. R package version 2.5 (2020).

17. C. R. Jackson, H. L. Tyler, J. J. Millar, Determination of microbial extracellular enzyme activity in waters, soils, and sediments using high throughput microplate assays. *J. Vis. Exp.* **80**, 1–9 (2013).

18. I. Cordero, H. Snell, R. D. Bardgett, High throughput method for measuring urease activity in soil. *Soil Biol. Biochem.* **134**, 72–77 (2019).

19. R. L. Sinsabaugh, A. E. Linkins, Exoenzyme activity associated with lotic epilithon. *Freshw. Biol.* **20**, 249–261 (1988).

20. J. L. DeForest, The influence of time, storage temperature, and substrate age on potential soil enzyme activity in acidic forest soils using MUB-linked substrates and l-DOPA. *Soil Biol. Biochem.* **41**, 1180–1186 (2009).

21. J. Rhymes, *et al.*, Are researchers following best storage practices for measuring soil biochemical properties? *SOIL* **7**, 95–106 (2021).

22. S. Kuo, "Phosphorus" in *Methods of Soil Analysis, Part 3. Chemical Methods*, D. L. Sparks, A. L. Page, P. A. Helmke, R. H. Loeppert, Eds. (Soil Science Society of America, American Society of Agronomy, 1996), pp. 869–919.

23. P. C. Brookes, A. Landman, G. Pruden, D. S. Jenkinson, Chloroform fumigation and the release of soil nitrogen: A rapid direct extraction method to measure microbial biomass nitrogen in soil. *Soil Biol. Biochem.* **17**, 837–842 (1985).

24. E. D. Vance, P. C. Brookes, D. S. Jenkinson, An extraction method for measuring soil microbial biomass C. *Soil Biol. Biochem.* **19**, 703–707 (1987).

25. G. P. Sparling, C. W. Feltham, J. Reynolds, A. W. West, P. Singleton, Estimation of soil microbial C by a fumigation extraction method: use on soils of high organic matter content, and a reassessment of the k_{EC}-factor. *Soil Biol. Biochem.* **22**, 301–307 (1990).

26. B. L. Turner, T. E. Romero, Stability of hydrolytic enzyme activity and microbial phosphorus during storage of tropical rain forest soils. *Soil Biol. Biochem.* **42**, 459–465 (2010).

27. P. C. Brookes, D. S. Powlson, D. S. Jenkinson, Measurement of microbial biomass phosphorus in soil. *Soil Biol. Biochem.* **14**, 319–329 (1982).

28. J. Rousk, E. Bååth, Growth of saprotrophic fungi and bacteria in soil. *FEMS Microbiol. Ecol.* **78**, 17–30 (2011).

29. E. Bååth, Measurement of protein synthesis by soil bacterial assemblages with the leucine incorporation technique. *Biol. Fertil. Soils* **17**, 147–153 (1994).

30. E. Bååth, Estimation of fungal growth rates in soil using ^{14}C-acetate incorporation into ergosterol. *Soil Biol. Biochem.* **33**, 2011–2018 (2001).

31. E. A. de Nijs, L. C. Hicks, A. Leizeaga, A. Tietema, J. Rousk, Soil microbial moisture dependences and responses to drying–rewetting: the legacy of 18 years drought. *Glob. Chang. Biol.* **25**, 1005–1015 (2019).

32. _ R Core Team, *R: a language and environment for statistical computing* (R Foundation for Statistical Computing, 2020).

33. P. J. McMurdie, S. Holmes, Waste not, want not: why rarefying microbiome data is inadmissible. *PLoS Comput. Biol.* **10**, e1003531 (2014).

34. A. D. Willis, Rarefaction, alpha diversity, and statistics. *Front. Microbiol.* **10**, 2407 (2019).

35. A. Willis, *et al.*, breakaway: Species Richness Estimation and Modeling. R package version 4.7.3 (2020).

36. A. D. Willis, B. D. Martin, Estimating diversity in networked ecological communities. *Biostatistics* (2020).

37. M. I. Love, W. Huber, S. Anders, Moderated estimation of fold change and dispersion for RNA-seq data with DESeq2. *Genome Biol.* **15**, 1–21 (2014).

38. M. E. Ritchie, *et al.*, *limma* powers differential expression analyses for RNA-sequencing and microarray studies. *Nucleic Acids Res.* **43**, e47 (2015).

39. N. H. Nguyen, *et al.*, FUNGuild: An open annotation tool for parsing fungal community datasets by ecological guild. *Fungal Ecol.* **20**, 241–248 (2016).

40. M. De Cáceres, P. Legendre, Associations between species and groups of sites: indices and statistical inference. *Ecology* **90**, 3566–3574 (2009).

41. P. J. Mcmurdie, S. Holmes, phyloseq: an R package for reproducible interactive analysis and graphics of microbiome census data. *PLoS One* **8**, e61217 (2013).

42. J. N. Paulson, O. Colin Stine, H. Corrada Bravo, M. Pop, Differential abundance analysis for microbial marker-gene surveys. *Nat. Methods* **10**, 1200–1202 (2013).

43. V. Mikryukov, metagMisc: Miscellaneous functions for metagenomic analysis (2020).

44. F. T. Maestre, *et al.*, Plant species richness and ecosystem multifunctionality in global drylands. *Science (80-.).* **335**, 214–218 (2012).

45. C. Wagg, S. F. Bender, F. Widmer, M. G. A. Van Der Heijden, Soil biodiversity and soil community composition determine ecosystem multifunctionality. *Proc. Natl. Acad. Sci. U. S. A.* **111**, 5266–5270 (2014).

46. J. Pinheiro, D. Bates, S. DebRoy, D. Sarkar, R. C. Team, nlme: Linear and Nonlinear Mixed Effects Models. R package version 3.1-149 (2020).

47. Y. Benjamini, Y. Hochberg, Controlling the false discovery rate: a practical and powerful approach to multiple testing. *J. R. Stat. Soc. Ser. B* **57**, 289–300 (1995).

48. M. Jafari, N. Ansari-Pour, Why, when and how to adjust your P values? *Cell J.* **20**, 604–607 (2019).

Table S1. Main soil chemical characteristics. Variables measured after pot experiment setup but before drought treatments. Mean ± standard deviation are shown (n=4). OM: organic matter content measured by loss on ignition. DOC: dissolved organic carbon, DON: dissolved organic nitrogen, TOP: total organic phosphorus, C_{mic}: microbial carbon, N_{mic}: microbial nitrogen, P_{mic}: microbial phosphorus.

**Variable**	**Value**
pH	6.11 ± 0.08
OM (%)	8.89 ± 0.07
Plant available NH_4^+ (mg Kg^{-1} dry soil)	8.09 ± 1.28
Plant available NO_3^- (mg Kg^{-1} dry soil)	6.20 ± 1.40
Plant available PO_4^{3-} (mg Kg^{-1} dry soil)	1.83 ± 0.64
DOC (mg Kg^{-1} dry soil)	49.28 ± 4.45
DON (mg Kg^{-1} dry soil)	5.52 ± 1.73
TOP (mg Kg^{-1} dry soil)	556.37 ± 58.29
C_{mic} (mg Kg^{-1} dry soil)	1348.4 ± 259.4
N_{mic} (mg Kg^{-1} dry soil)	172.0 ± 35.9
P_{mic} (mg Kg^{-1} dry soil)	182.5 ± 34.7

Table S2. Effects of drought intensity, drought frequency, and harvest time point on soil bacterial and fungal taxa, analysed by linear mixed models with soil as random factor. F values and significance levels are given as *<0.05; **<0.01; ***<0.001; ^{ns}: not significant.

	Intensity (I)	Frequency (F)	Time point (T)	I x F	I x T	F x T	I x F x T
Bacterial Phylum							
Proteobacteria	39.68 ***	3.68 *	20.13 ***	1.14 ^{ns}	11.71 ***	0.49 ^{ns}	0.24 ^{ns}
Acidobacteriota	120.61 ***	1.85 ^{ns}	35.69 ***	0.96 ^{ns}	4.71 **	1.25 ^{ns}	1.58 ^{ns}
Verrucomicrobiota	1.99 ^{ns}	2.95 ^{ns}	24.15 ***	1.41 ^{ns}	0.91 ^{ns}	3.10 *	1.33 ^{ns}
Actinobacteriota	3.58 *	4.06 *	2.40 ^{ns}	0.63 ^{ns}	6.81 ***	1.22 ^{ns}	0.94 ^{ns}
Firmicutes	11.45 ***	0.46 ^{ns}	4.04 *	1.20 ^{ns}	1.08 ^{ns}	0.76 ^{ns}	0.56 ^{ns}
Myxococcota	206.27 ***	2.28 ^{ns}	347.20 ***	3.52 *	24.21 ***	1.82 ^{ns}	1.46 ^{ns}
Bacteroidota	61.64 ***	1.54 ^{ns}	62.34 ***	0.79 ^{ns}	4.47 **	1.19 ^{ns}	0.94 ^{ns}
Planctomycetota	0.28 ^{ns}	1.92 ^{ns}	102.26 ***	0.16 ^{ns}	1.56 ^{ns}	0.32 ^{ns}	2.34 *
Chloroflexi	12.12 ***	1.94 ^{ns}	1.29 ^{ns}	0.47 ^{ns}	10.20 ***	1.61 ^{ns}	0.70 ^{ns}
Fungal Phylum							
Ascomycota	16.46 ***	6.24 ^{ns}	0.07 ^{ns}	5.73 ***	1.72 ^{ns}	0.36 ^{ns}	0.75 ^{ns}
Basidiomycota	4.27 *	2.06 ^{ns}	3.46 *	1.63 ^{ns}	0.52 ^{ns}	1.22 ^{ns}	0.24 ^{ns}
Mortierellomycota	9.77 ***	1.41 ^{ns}	1.44 ^{ns}	3.03 *	5.27 ***	1.14 ^{ns}	1.03 ^{ns}
Chytridiomycota	8.18 ***	0.30 ^{ns}	5.56 **	0.36 ^{ns}	0.10 ^{ns}	3.05 *	1.21 ^{ns}

Table S3. Effects of drought on soil extracellular enzyme activities and soil nutrients analysed by linear mixed models with drought intensity (I) and frequency (F) as fixed factors and soil as random factor. It is also shown if transformation to meet model assumptions was needed (Ln = natural logarithm transformation, Sqrt= square root transformation). Significant results are highlighted in bold. GLC: *β*-glucosidase, CBH: cellobiohydrolase, XYL: xylosidase, NAG: *N*-acetylglocasiminidase, PHO: acid phosphatase, URE: urease, POX: phenoloxidase, PER: peroxidase, DOC: dissolved organic carbon, DON: dissolved organic nitrogen, TOP: total organic phosphorous, C_{mic}: microbial carbon, N_{mic}: microbial nitrogen, P_{mic}: microbial phosphorus.

	After drought (t1)							Six months after drought (t4)						
**Variable**	**Intensity (I)**		**Frequency (F)**		**IxF**			**Intensity (I)**		**Frequency (F)**		**IxF**		
	F	p	F	p	F	p		F	p	F	p	F	p	
GLC	67.58	**<0.001**	8.62	**0.002**	8.19	**<0.001**		50.44	**<0.001**	0.83	0.449	0.44	0.779	
CBH	44.29	**<0.001**	5.07	**0.015**	6.18	**0.002**		49.93	**<0.001**	2.31	0.121	0.95	0.455	
XYL	49.03	**<0.001**	1.94	0.166	5.01	**0.004**		21.47	**<0.001**	1.06	0.363	0.43	0.787	
NAG	34.43	**<0.001**	13.60	**<0.001**	6.85	**<0.001**		19.13	**<0.001**	1.12	0.342	1.18	0.344	
PHO	32.69	**<0.001**	20.20	**<0.001**	5.70	**0.002**		8.03	**0.002**	11.43	**<0.001**	0.43	0.782	
URE	0.28	0.757	3.51	**0.046**	3.31	**0.027**		35.91	**<0.001**	0.61	0.554	0.94	0.451	
POX	0.14	0.872	2.84	0.078	0.88	0.493		0.54	0.589	1.59	0.226	0.35	0.840	
PER	0.90	0.419	5.34	**0.012**	1.61	0.203		0.13	0.877	2.85	0.078	1.81	0.160	
NH_4^+	165.39	**<0.001**	33.77	**<0.001**	7.39	**<0.001**		23.35	**<0.001**	2.51	0.102	0.58	0.683	
NO_3^-	1.00	0.382	2.18	0.135	2.40	0.078		7.45	**0.003**	0.14	0.871	2.33	0.085	
PO_4^{3-}	17.09	**<0.001**	6.12	**0.007**	0.91	0.472		1.36	0.274	0.98	0.391	0.28	0.889	
DOC	38.76	**<0.001**	3.81	**0.037**	1.56	0.219	Ln	1.32	0.285	1.03	0.371	0.94	0.459	
DON	16.68	**<0.001**	0.14	0.867	1.55	0.219	Ln	0.17	0.848	0.27	0.765	1.37	0.274	
TOP	0.06	0.945	0.02	0.983	0.27	0.892		2.58	0.096	0.13	0.882	1.33	0.286	
C_{mic}	15.66	**<0.001**	4.02	**0.031**	2.38	0.080		7.87	**0.002**	0.77	0.474	0.88	0.493	
N_{mic}	22.02	**<0.001**	0.77	0.474	1.79	0.164		12.34	**<0.001**	0.06	0.944	0.93	0.464	
P_{mic}	15.86	**<0.001**	2.38	0.114	0.75	0.570		42.82	**<0.001**	0.43	0.654	1.18	0.343	Sqrt
pH	20.42	**<0.001**	5.43	**0.011**	2.20	0.099		11.10	**<0.001**	0.63	0.542	1.30	0.297	

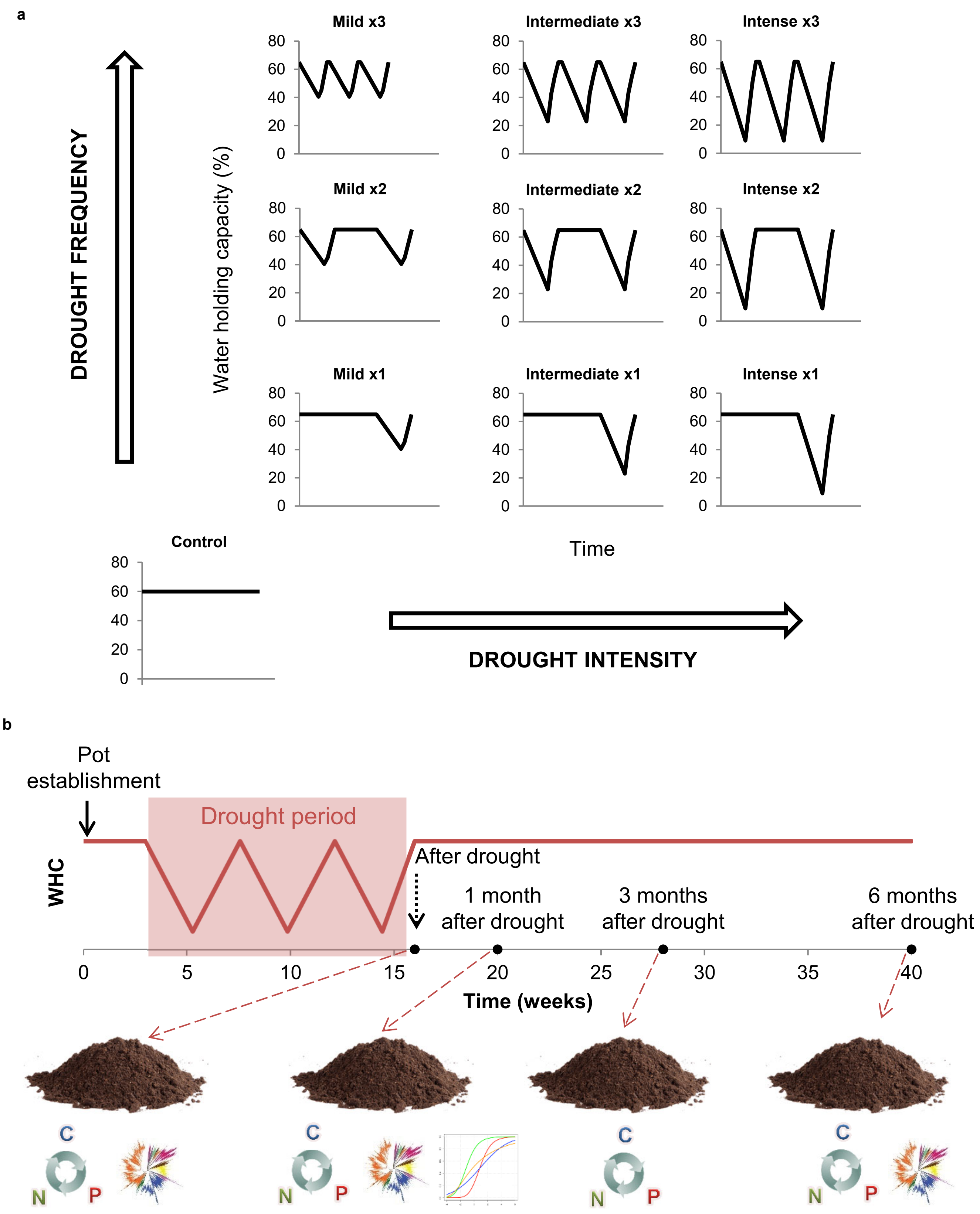

Fig. S1 Experimental design summary. a, Schematic representation of the different drought levels, including 3 intensity levels, 3 frequency levels, and a control soil. **b**, Timeline showing the start of the experiment, the drought period and the harvests after drought. The different measurements done at each individual time point are represented as: a C,N,P cycle = soil nutrient pools and enzymes, a phylogenetic tree = fungal and bacterial DNA sequencing, and growth curves = microbial growth and respiration rates.

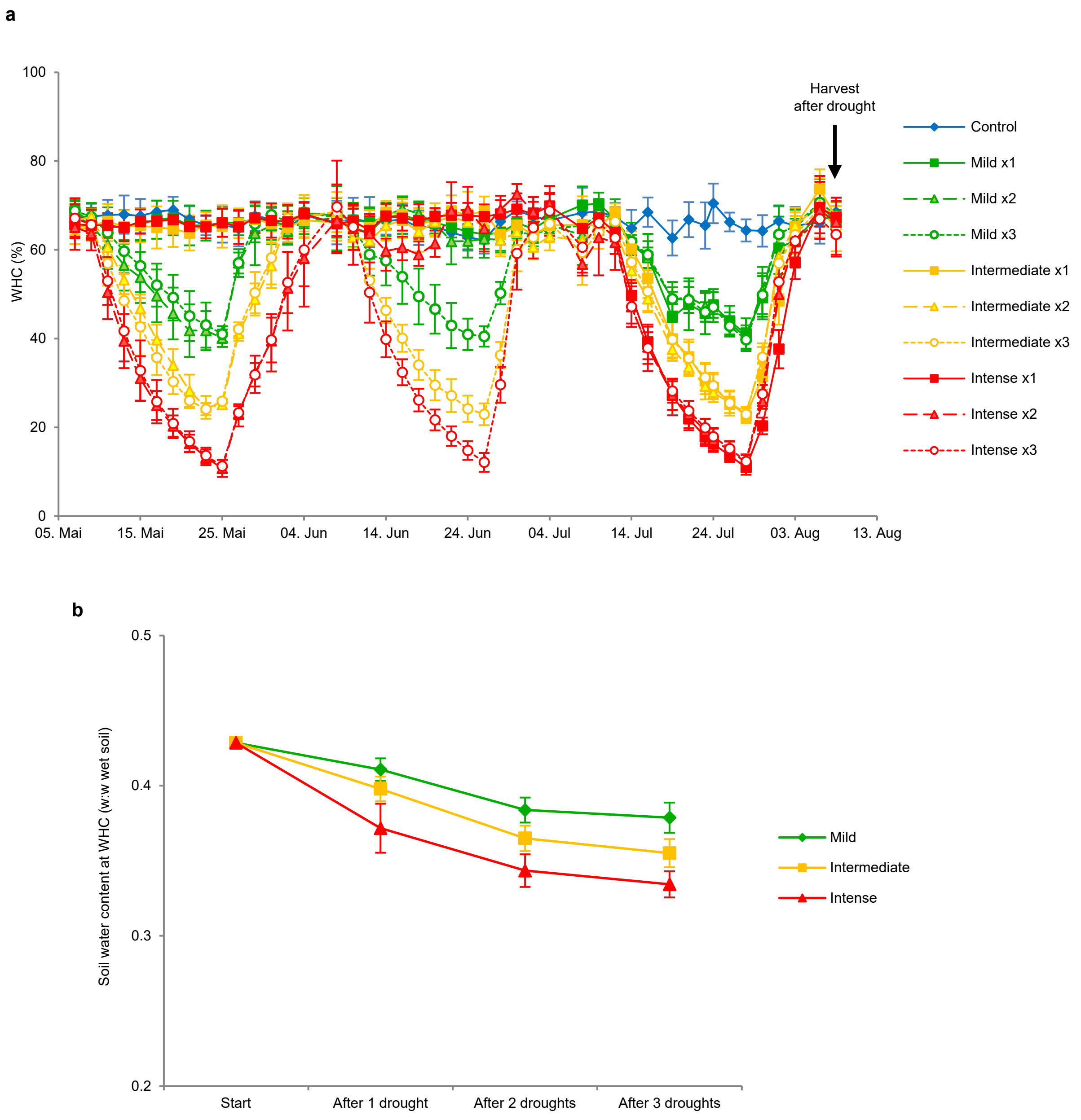

Fig. S2 Soil water holding capacity (WHC). a, Evolution of WHC along the experimental period depending on drought intensity (I) and frequency (F). The arrow indicates the date of the harvest after drought. **b,** Effects of drought on WHC of soils. Values = mean ± SD (n= 4).

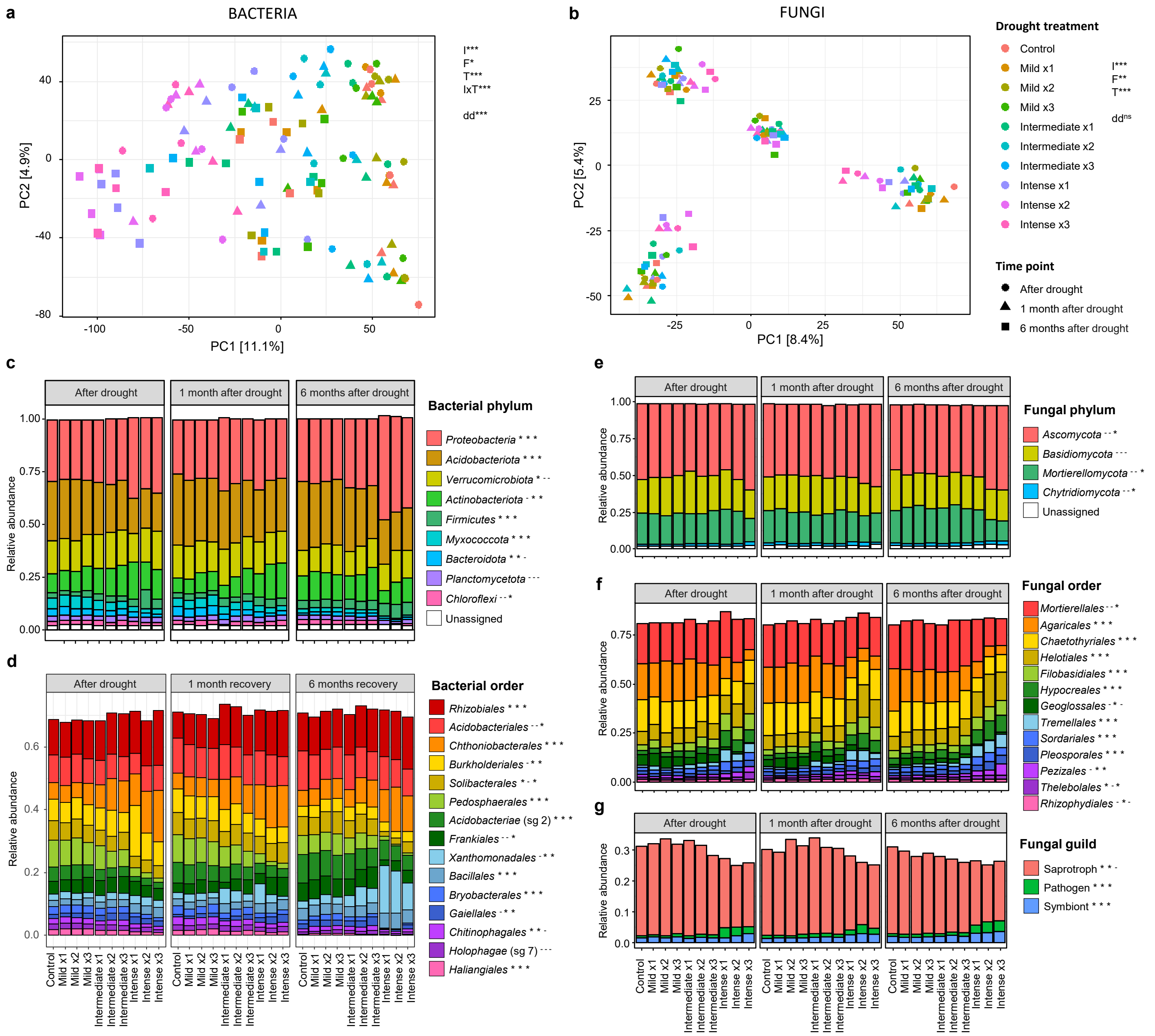

Fig. S3 Microbial community structure. Data based on 16S (bacteria) and ITS2 (fungi) amplicon sequencing an summarised by drought intensity and frequency (x1: 1 event, x2: 2 events, x3: 3 events) treatments. **a,b,** Community structure evaluated by principal component analysis of VST transformed data, without removing soil effect. Significance of PERMANOVA analysis evaluating the effects of drought intensity (I), frequency (F) and sampling time (T), with soil as random factor, is shown in each graph: *p<0.05, **p<0.01, ***p<0.001. Differences in data dispersion (dd) among groups is also shown (ns: non-significant). Variance explained by soil is 9.9% for bacteria (**a**) and 18.4% for fungi (**b**). **c-g**, Relative abundance of different microbial taxa depending on drought treatment and sampling time. Data = mean of 4 replicates. Only taxa with relative abundances >1% across all samples are plotted. Fungal guild data extracted from FUNGuild database. Significance of linear mixed models evaluating the effects of drought treatment for each sampling time with soil as random factor, is shown to the right of each taxa name as - p>0.05,*p<0.05. sg = subgroup.

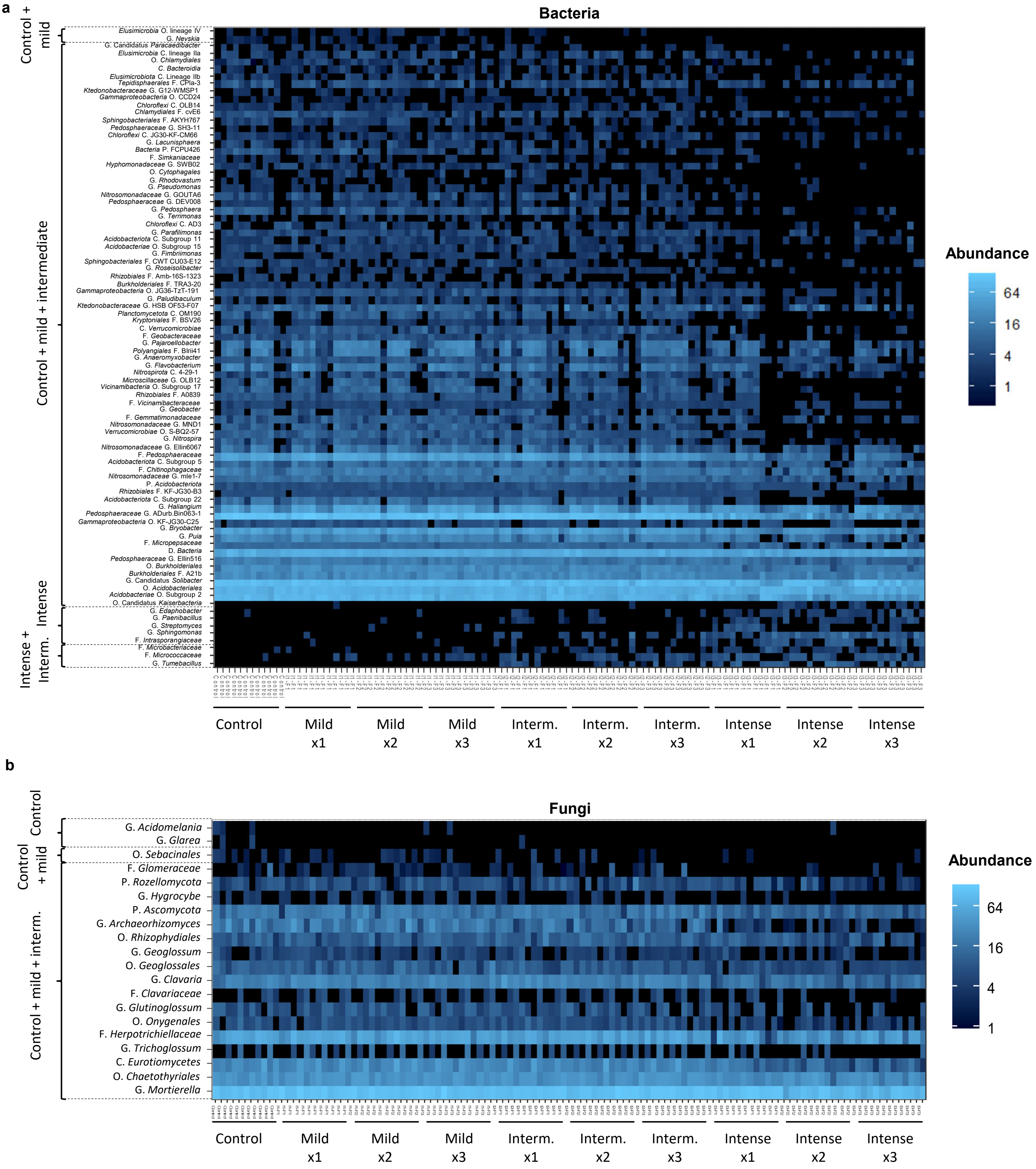

Fig. S4 Indicator species analysis. Heatmaps showing cumulative sum scaling (CSS) normalised abundances of soil bacteria (**a**) and fungi (**b**) indicator taxa identified for different drought intensity and frequency (x1: 1 event, x2: 2 events, x3: 3 events) treatments. Only taxa significant at $p \leq 0.001$ (bacteria) or $p \leq 0.01$ (fungi) are shown. Data agglomerated at genus level for analysis. ASVs unassigned at genus level, show the name of the lowest assigned taxonomical rank (P=Phylum, C=Class, O=Order, F=Family, G=Genus). Interm. = intermediate.

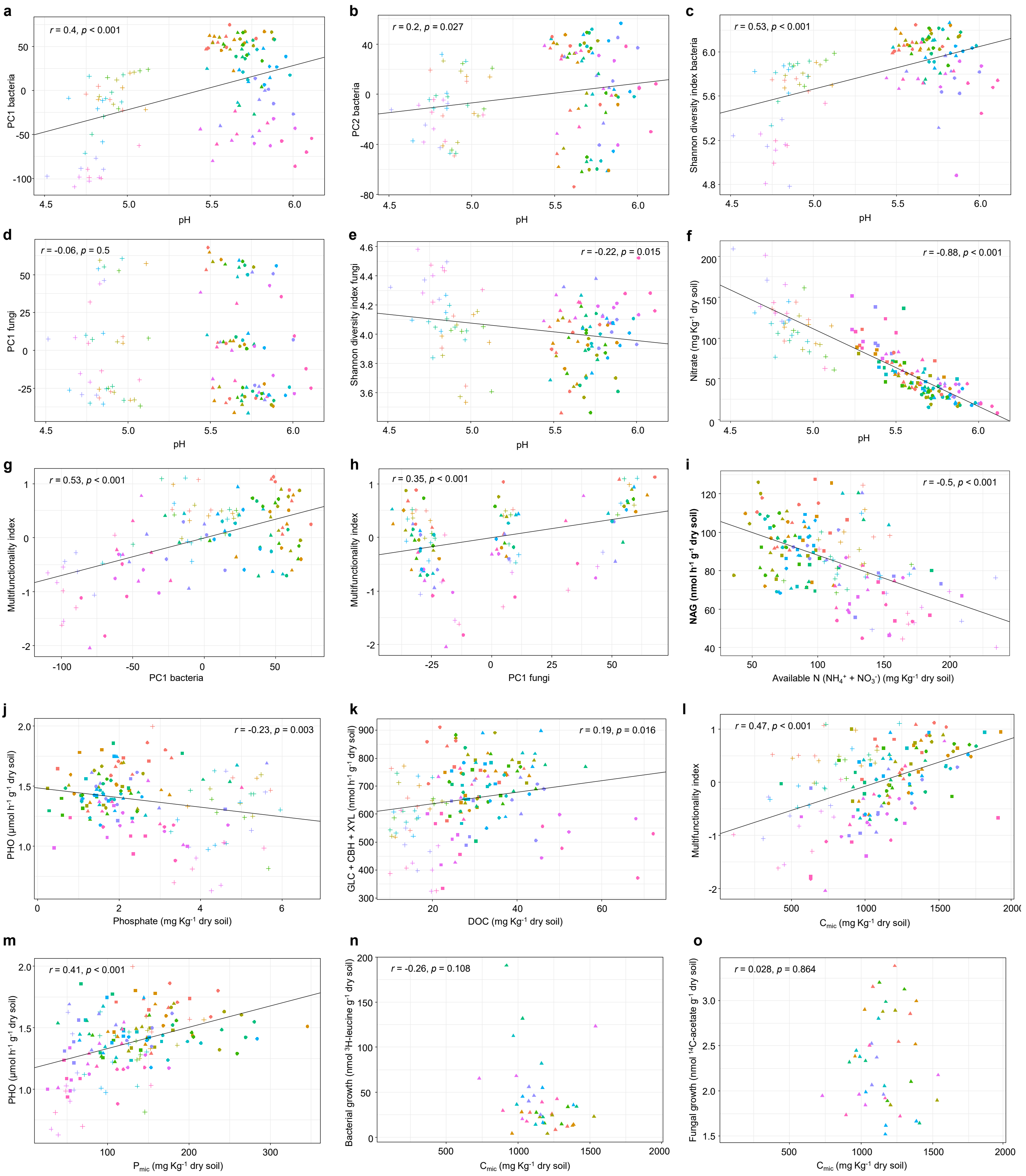

Fig. S5 Correlations between soil chemical and biological variables. Pearson correlation value and significance test are shown in each graph. Regression line is represented when the correlation is significant ($p < 0.05$). GLC: *β*-glucosidase, CBH: cellobiohydrolase, XYL: xylosidase, NAG: *N*-acetylglocasiminidase, PHO: acid phosphatase, C_{mic}: microbial carbon, N_{mic}: microbial nitrogen, P_{mic}: microbial phosphorus. PCA axis correspond to the microbial community structure analyses in Extended Data Fig. 1. Multifunctionality index represents a combined index of all analysed enzyme activities.

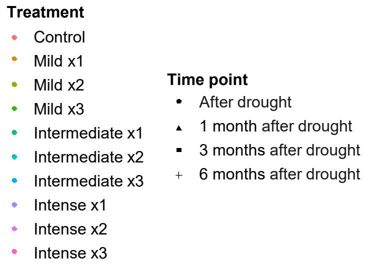

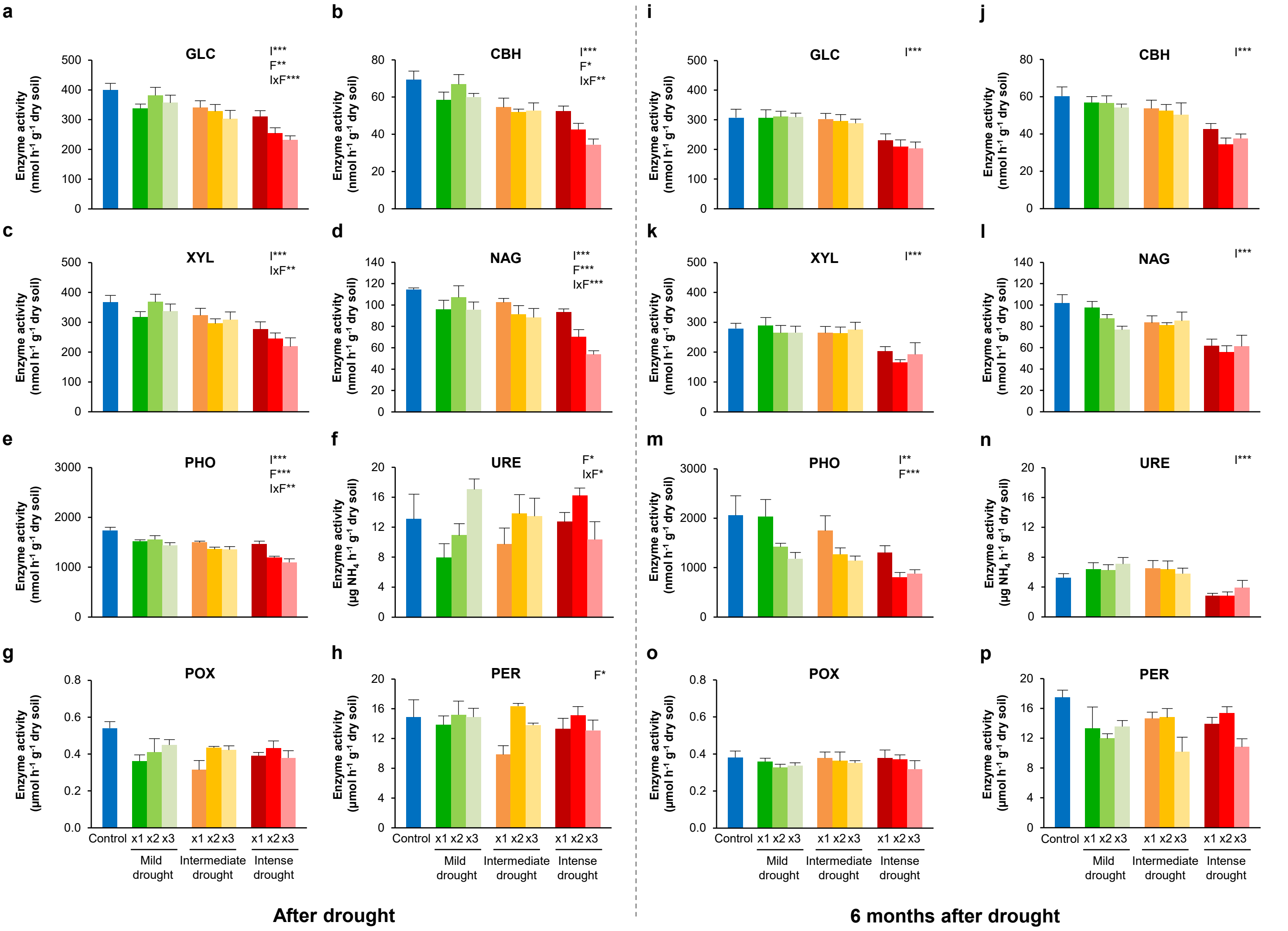

Fig. S6 Effects of drought on soil enzyme activities. Data after drought (**a-h**) and after 6 months afterwards (**i-p**), summarised by drought intensity and frequency (x1: 1 event, x2: 2 events, x3: 3 events) treatments. Significance of linear mixed models evaluating the effects of drought intensity (I) and frequency (F), with soil as random factor, is shown in each graph: *p<0.05, **p<0.01, ***p<0.001. Values = mean ± standard error, n=4. GLC: *β*-glucosidase, CBH: cellobiohydrolase, XYL: xylosidase, NAG: *N*-acetylglocasiminidase, PHO: acid phosphatase, URE: urease, POX: phenoloxidase, PER: peroxidase.

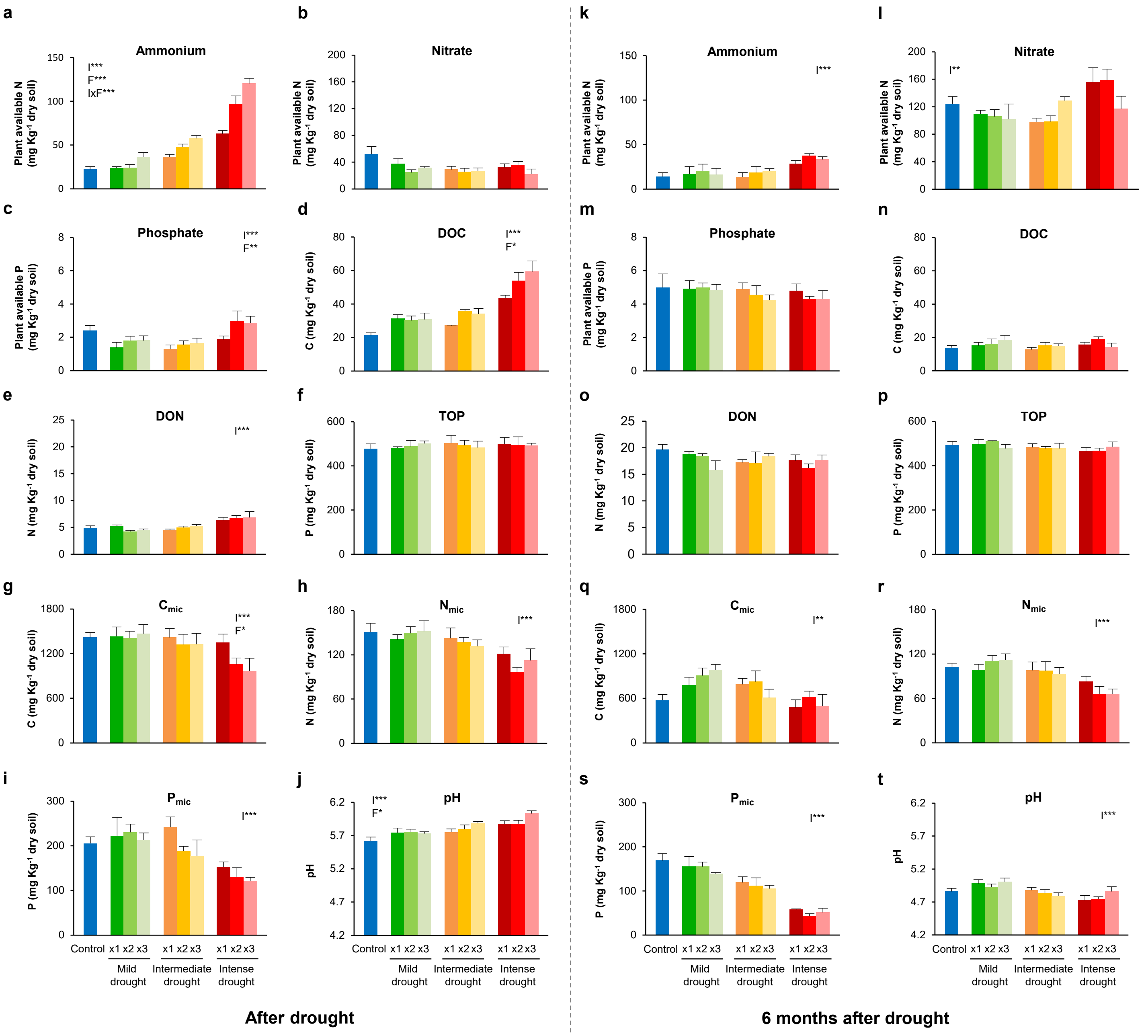

Fig. S7 Effects of drought on soil nutrients, microbial biomass and pH. Data after drought (**a-j**) and after 6 months afterwards (**k-t**), summarised by drought intensity and frequency (x1: 1 event, x2: 2 events, x3: 3 events) treatments. Significance of linear mixed models evaluating the effects of drought intensity (I) and frequency (F), with soil as random factor, is shown in each graph: *p<0.05, **p<0.01, ***p<0.001. Values = mean ± standard error, n= 4. DOC: dissolved organic carbon, DON: dissolved organic nitrogen, TOP: total organic phosphorous, C_{mic}: microbial carbon, N_{mic}: microbial nitrogen, P_{mic}: microbial phosphorus.

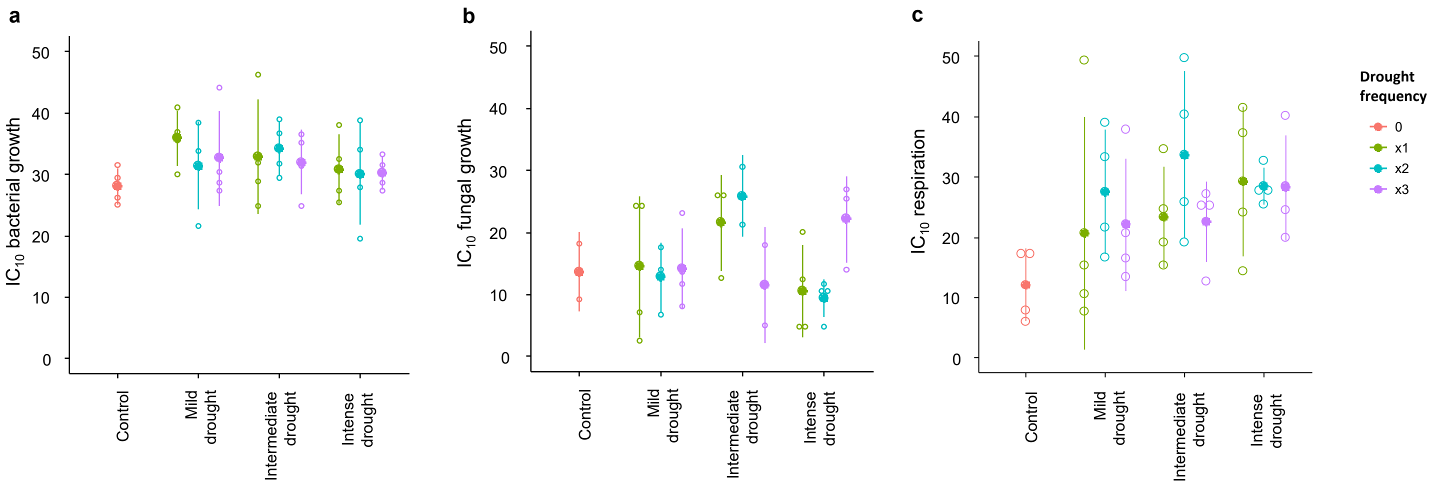

Fig. S8 Moisture dependence of bacterial and fungal growth and respiration depending on drought history. IC_{10} represents the moisture level (in % water holding capacity) at which growth and respiration rates are reduced by 10%. Drought intensity and drought frequency had no significant effects on these variables. Drought frequency x1=1 event, x2=2 events, x3=3 events. Mean ± SD (n=4) and individual data points shown as open circles.

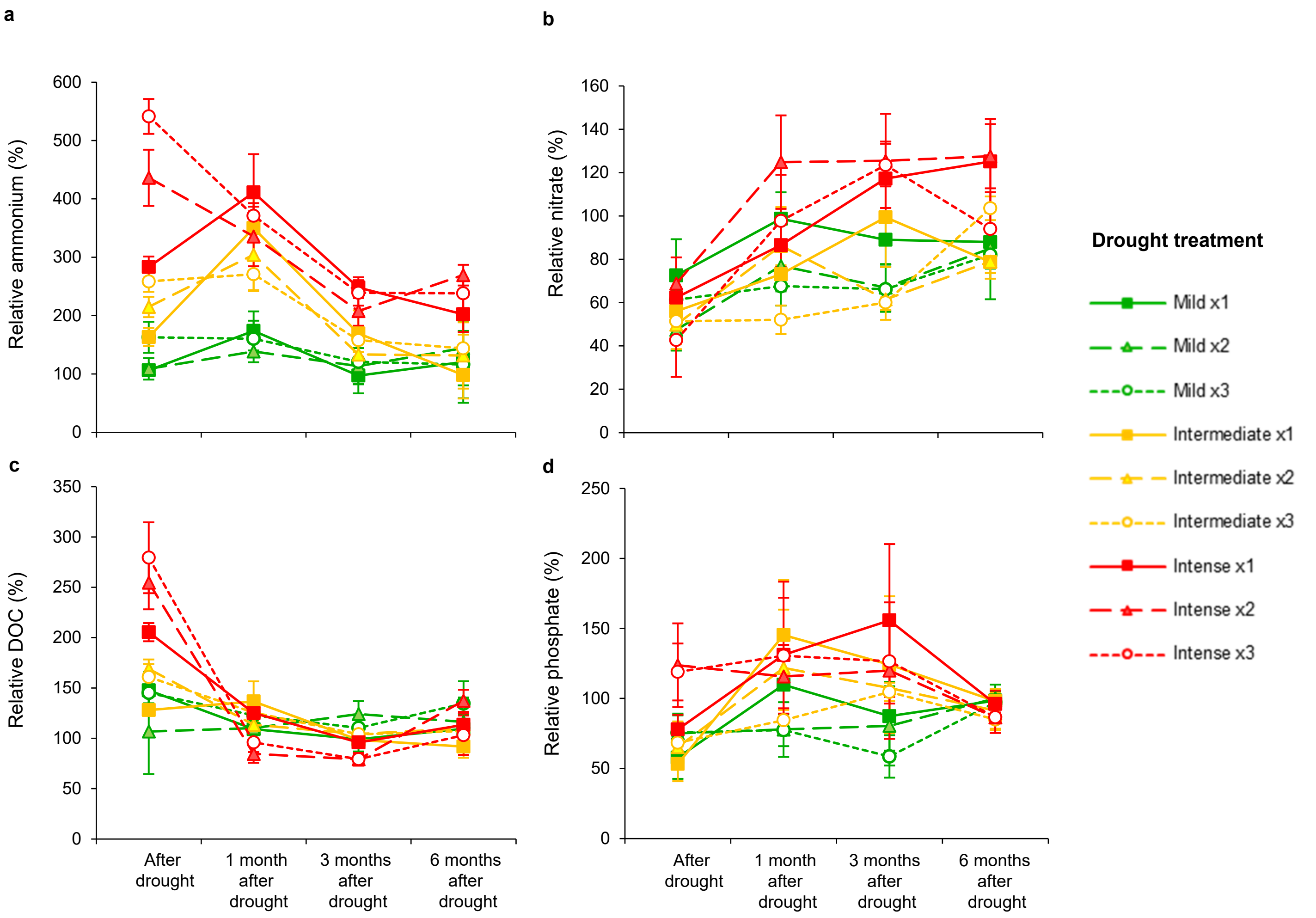

Fig. S9 Evolution over time of nutrient contents in soils depending of the drought treatment, relative to the control (control value = 100%). Values = mean ± standard error, n= 4. DOC: dissolved organic carbon. Drought frequency (x1: 1 event, x2: 2 events, x3: 3 events).
